# Clinical Reasoning Uncertainty in Veterinary Medical Encounters with a Clinical Example

**DOI:** 10.3390/vetsci12121203

**Published:** 2025-12-15

**Authors:** Kiro Risto Petrovski, Roy Neville Kirkwood

**Affiliations:** 1 School of Animal and Veterinary Sciences, The University of Adelaide, Roseworthy, SA 5371, Australia; 2 Davies Livestock Research Centre, School of Animal and Veterinary Sciences, The University of Adelaide, Roseworthy, SA 5371, Australia

**Keywords:** ambiguity, clinical judgment, clinical activities, clinical practice, metacognition, teaching, veterinary learners, work-based learning

## Abstract

This narrative review examines the complexities of medical uncertainty in veterinary practice, highlighting its significant implications for clinical reasoning and decision-making. Veterinary professionals face inherent uncertainties due to factors such as biological variability, incomplete knowledge, and the pressures of rapidly evolving practices. The distinction between clinical ambiguity and medical uncertainty is crucial, as it informs the coping strategies employed by veterinarians. While uncertainty is often viewed negatively, it can stimulate curiosity and enhance problem-solving capabilities. This review categorizes uncertainty into aleatoric and epistemic types, offering insights into their origins and impacts on veterinary professionals and client interactions. The dynamic nature of uncertainty influences both immediate clinical encounters and long-term professional development, with varying effects based on individual tolerance levels and situational stakes. Despite the growing body of literature on uncertainty, veterinary education often neglects to address this critical aspect, leading to a gap in metacognitive competencies among practitioners. We have included a case example that offers explicit guidelines on the application of the Five Microskills model of clinical teaching. This model is proposed to assist veterinary professionals in effectively managing uncertainty. To enhance the quality of veterinary care, there is an urgent need to integrate uncertainty management into veterinary curricula and ongoing professional development. By fostering an environment that acknowledges and addresses uncertainty, veterinary professionals can improve their clinical reasoning, strengthen client relationships, and ultimately enhance patient outcomes. This review advocates for the adoption of evidence-based practices and collaborative approaches to navigate the complexities of uncertainty, ensuring high standards of care in veterinary medicine.

## Simple Summary

In veterinary practice, professionals often face challenges when making diagnoses and treatment plans due to the inherent uncertainties in their field. These uncertainties can arise from various factors, including the complexity of animal health, a lack of complete knowledge, and the unpredictability of cases. Understanding the difference between clinical ambiguity—where multiple hypotheses seem valid—and medical uncertainty—where veterinarians lack confidence in their knowledge—is crucial. Uncertainty can negatively impact decision-making, leading to errors and biases, but it can also encourage curiosity and problem-solving. Despite the importance of managing uncertainty, veterinary education often neglects to teach coping strategies, which are usually learned through experience. This review emphasizes the need for veterinary professionals to develop strategies for dealing with uncertainty, as it can influence their mental health and the quality of care they provide. By enhancing education and communication about uncertainty, veterinarians can improve their practice and client relationships.

## 1. Introduction

In veterinary practice, professionals strive to reach a final diagnosis and management plan. However, achieving a definitive diagnosis is not always possible, and multiple treatment paths may exist for various cases [1,2,3]. Clinical ambiguity arises when several hypotheses seem equally valid, while medical uncertainty occurs when a veterinary professional lacks the knowledge or understanding necessary for confident clinical reasoning [4,5,6]. It is important to distinguish between ambiguity and uncertainty as defined by social scientists [7].

Clinical ambiguity and medical uncertainty are inherent in veterinary practice [8], alongside educational uncertainties regarding what needs to be learned for clinical practice and assessments, as well as professional [9,10,11,12,13] and technological [14] uncertainties due to rapidly evolving practices. Additionally, uncertainty in veterinary practice may occur due to other factors (e.g., exposure to unfamiliar animal species) [8,14]. This paper focuses on medical uncertainty, which is often blamed for errors in clinical reasoning [15,16,17]. However, uncertainty can also be viewed positively, stimulating curiosity, or be inconsequential if it does not significantly impact decision-making [18].

People intrinsically differ in their tolerance to uncertainty [2,5,8,12,15,19,20,21,22,23,24,25,26,27,28,29,30,31,32,33,34,35,36], but tolerance for uncertainty is crucial, influencing biases and errors in reasoning [15,16,19,21,36,37]. Uncertainty is a metacognitive competence that significantly affects a veterinary professional’s clinical reasoning competency [12,38,39,40]. Despite its importance, metacognitive competencies related to uncertainty are frequently neglected in veterinary education, and in practice, are often learned informally through experience [29,41,42]. Veterinary professionals must be equipped to handle both clinical reasoning and various uncertainties [3,17,22,42,43,44,45]. Awareness of concepts, implications, and coping strategies is vital for effectively managing uncertainty [8,43,46].

Despite over eight decades of literature on uncertainty [12], a widely accepted definition remains elusive [5,8,11,12,19,25,26,46,47,48], leading to significant variability in the field, including the methodology for measuring its prevalence [11,26,32,33,46,49,50,51,52]. Changing public perception over time further complicates this issue [11,15,25,26,32,46,53]. Increased public awareness of the limitations of medical knowledge, fueled by media coverage and movements advocating for evidence-based veterinary medicine, has prompted clients to engage more actively in their animals’ healthcare decisions. As a result, veterinary professionals may need to adapt their communication strategies to meet these evolving expectations [54,55].

The complexity of measuring uncertainty is compounded by the belief that it equates to failure, leading to nondisclosure and impacting its perceived prevalence [56,57]. Uncertainty has both short-term and long-term effects on veterinary practice, yet research primarily focuses on the former [44]. A holistic approach to understanding the causes and outcomes of uncertainty is rare.

The categorization of uncertainty is very confusing [11,12,18,58] and has been the subject of enduring argument in medical, philosophical and social science literature. We categorize uncertainty in veterinary medicine into three types: (a) Aleatoric uncertainty, resulting from biological variability and unreliable sampling (e.g., there is a 50% probability of achieving a cure rate for subclinical mastitis caused by *Staphylococcus aureus* in dairy cows treated with a dry cow therapy antimicrobial product. This probability is contingent upon several factors, including the age of the cow, the duration of the intramammary infection, the period during which the antimicrobial concentration remains above the minimum inhibitory concentration, the number of infected quarters, and the cow’s prior history of mastitis); (b) Epistemic uncertainty, arising from incomplete understanding of veterinary conditions (e.g., explanation of pathophysiology for some veterinary disorders; mental organization of the knowledge by the veterinary professional)–NOTE: Some say this is medical ambiguity; and (c) a combination of both, which represents a combination of both approaches, which is frequently encountered in clinical settings, particularly in relation to system-related uncertainty that will be addressed in subsequent sections [5,12,19,39,59].

Tolerance for uncertainty directly influences clinical reasoning capabilities in veterinary professionals [11,15,32,33,40,46]. Coping with uncertainty is a core competency in both human [60,61,62] and veterinary [33,63,64,65] medicine. However, the teaching of uncertainty in veterinary education is not standardized, often relying on informal methods [8,66,67]. This leaves both learners and instructors without clear advice on how to best learn or teach.

**Figure 1:**
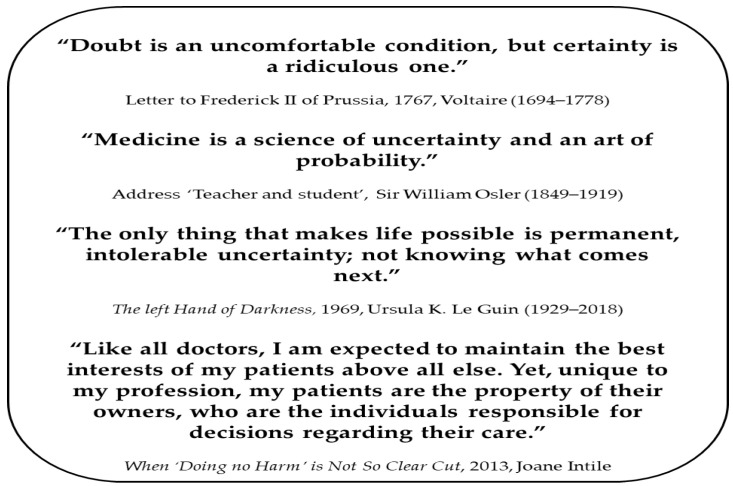
Famous quotes related to medical and veterinary practice uncertainties.

In this narrative review, we explore the concepts of uncertainty, common causes, and their effects on veterinary professionals. We also address the recognition of individuals experiencing difficulties, the implications for service quality, and propose strategies for mitigation. Additionally, we highlight gaps in the veterinary literature, which frequently draws from human medical fields, and propose that the same principles can be applied to veterinary practice. However, it is important to acknowledge certain distinctions. For instance, the veterinary medical field faces uncertainties that are unique to the profession: 1. Patients may belong to various species, each presenting specific uncertainties that do not have counterparts in human medicine; 2. The patient is owned or represented by individuals responsible for their well-being, which is somewhat analogous to the dynamics in pediatrics and intensive care units (see Figure 1). A significant proportion of the cited literature originates from human medical fields, including dentistry, emergency medicine, nursing, and oncology. In this paper, we assume that the same principles would apply to the veterinary medical field, but we know that much of the cited literature is either not veterinary in nature or is based on opinion rather than empirical research. 

The training needs related to uncertainty have been previously found to be similar for medical and veterinary professionals and educators [10]. Due to the variety of species seen by veterinary professionals, it has been postulated that they suffer from a higher level of uncertainty compared to medical practitioners [33]. Recognizing inherent uncertainties may lead veterinary professionals to adopt strategies for minimizing negative impacts (e.g., burnout, mental health issues) and maximizing potential benefits (e.g., curiosity, problem-solving capacity). By fostering this awareness, veterinarians can enhance decision-making, improve communication with clients, and promote better patient outcomes. Continuing education, collaboration, and evidence-based practices are essential for navigating the complexities of veterinary medicine and ensuring high standards of care. A case example addressing and teaching veterinary medical uncertainty is provided in the Supplementary Material.

**Figure 2:**
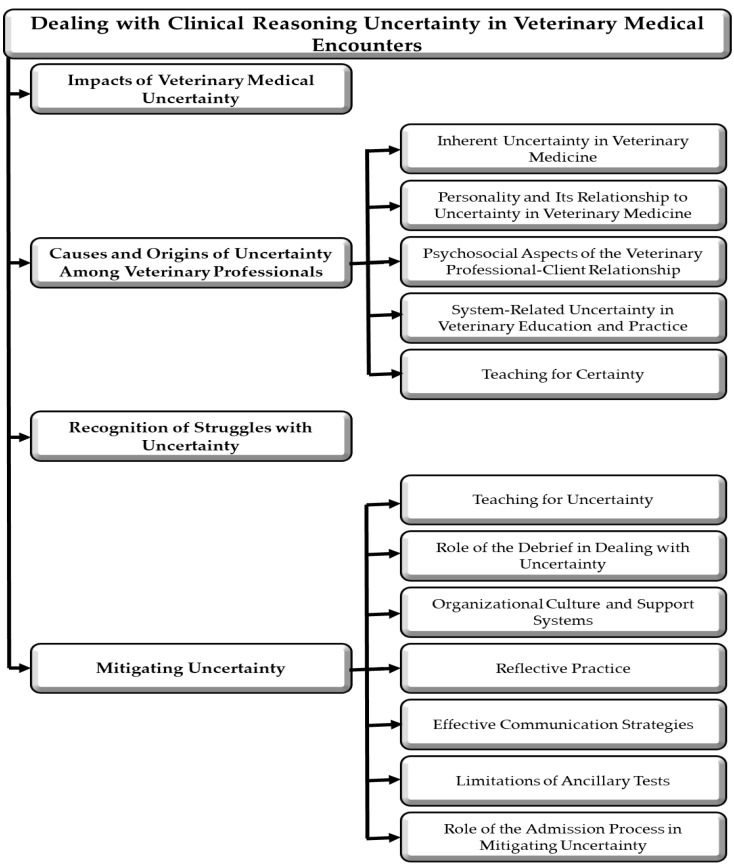
The structure of the body of this review.

The structure of the body of this review is presented in Figure 2. Key concepts and terminology are provided in the Glossary section at the conclusion of this paper. 

## 2. Search Methodology

For this narrative review, we conducted a comprehensive search using common electronic databases, including PubMed, Scopus, and Web of Science. We employed broad search terms such as “ambiguity,” “medical,” “uncertainty,” and “veterinary.” The following types of sources were deemed acceptable: commentaries, letters, original articles, and reviews (including narrative, scoping, and systematic reviews).

Only articles published in English from 1 January 1995 to mid-January 2025 were included. The databases were re-evaluated in early October 2025, leading to the addition of a few more articles. We included articles that addressed clinical reasoning competency but excluded those that focused solely on general uncertainty or where clinical reasoning competency was only marginally covered.

Our selection criteria encompassed articles from the fields of dentistry, medicine, nursing, and veterinary medicine. To identify relevant articles, we initially scanned titles and abstracts. Subsequently, the full texts of selected articles were reviewed for inclusion in this narrative review. Articles lacking full-text access were excluded from our analysis.

Data were extracted qualitatively and subsequently summarized for all included articles. In the following step, a synthesis of the summarized data was conducted. Where appropriate, data were organized into box, figure, or table format; otherwise, textual summaries were provided. Given that this is a narrative review, the authors did not adhere strictly to the recommendations for scoping reviews (e.g., ref. [68]) or systematic reviews (e.g., ref. [69]), although some principles were superficially applied. The authors also took into consideration the descriptors and guidelines pertinent to writing narrative reviews (e.g., refs. [70,71,72]).

## 3. Impacts of Veterinary Medical Uncertainty

Veterinary medical uncertainty can significantly impact clients, veterinary professionals, and the work environment, ±industry, especially concerning the performance of production animals (e.g., may affect the trade of live ruminants and their products when uncertainty of a reportable disease, such as foot-and-mouth disease, is present). Most reactions to uncertainty are maladaptive due to an inherent intolerance to uncertainty in humans [3,66]. The impact of uncertainty is dynamic [25,31,32] and varies based on its causes, the tolerance levels of both the clients and veterinary professionals, and the stakes involved in the encounter. Epistemic and psychosocial causes (discussed in subsequent sections; see Glossary) typically lead to greater impacts, particularly for individuals with a higher intolerance to uncertainty [21,25,73]. High-stakes situations, such as emergencies, euthanasia, or sudden death syndrome at a population level, tend to amplify these impacts [3,22,41,74,75].

While veterinary literature on this topic is limited [33], anecdotal evidence and empirical observations support many identified impacts presented in Table 1. Due to a significant lack of veterinary-specific literature on this matter, the authors have synthesized information from both medical and veterinary sources. The consequences of uncertainty can be behavioral, cognitive, and emotional [5,31,36], and it is essential to note that these areas are often interrelated (e.g., anxiety can lead to increased intolerance of uncertainty) [5,8,32,73]. The responses of veterinary professionals to uncertainty not only affect the immediate encounters but can also have long-lasting effects on their cognitive and metacognitive competencies and work–life balance [36,76].

Uncertainty can negatively influence clinical reasoning competency, making veterinary professionals more susceptible to biases [1,3,19,24,44,81], difficulties [3,81], and errors [3,24,81]. A common manifestation of this is “premature closure,” where decisions are made before all relevant information is considered [19,80,87,93]. Furthermore, anecdotally, veterinary professionals may hesitate to acknowledge or disclose uncertainty, hindering the expansion of their cognitive and metacognitive skills.

Emotional responses to uncertainty can create various competency-related issues. Modern veterinary practice is built on client-centered relationships, where open communication is critical. However, uncertainty can lead to professional fragility, causing veterinary professionals to withhold information [15,19,21,22,39,44,73,94], which undermines shared decision-making—an essential component of effective veterinary services.

In analogy to the medical profession, and primarily drawing from medical literature, the mental health of veterinary professionals may also be adversely affected by uncertainty, leading to short-term issues like anxiety [1,3,23,28,44,45,53,73,78,85,99,107] and stress [3,24,27,28,44,56,59,85,94], as well as long-term challenges [94] such as apathy, depression [1,21,44,53,76,99], denial [18,95], and sleep disturbances [21]. Mental health issues [1,3,9,21,22,23,25,43,76,85] and maladaptive coping strategies [1,3,9,21,23,24,27,44,53,66,73,85,91], particularly depression, are more prevalent among veterinary professionals compared to the general population [109,110,111], contributing to burnout and adverse outcomes, including suicidal ideation [33,35,66,84,95,99,102].

The negative effects of uncertainty can result in suboptimal veterinary services, contradicting the principle of *primum non nocere* (first, do no harm) [37]. Uncertainty may lead to avoidance, delayed decision-making, inaction, and inattention, resulting in frustration for both clients and professionals [29]. This frustration can strain the veterinary–client relationship, leading to changes in clinical behavior, such as reliance on intuition rather than deliberate decision-making (see Glossary). Additionally, uncertainty can result in resource wastage, including overprescribing, unnecessary referrals, and prolonged consultations, which can ultimately compromise animal welfare. In cases involving production animals, uncertainty can pose risks to trade and may even lead to unnecessary animal suffering (e.g., utilization of non-rational ancillary techniques, particularly when invasive).

Uncertainty in client expectations also negatively impacts the veterinary professional–client relationship [18]. It is crucial to ensure clients have access to quality veterinary care and to engage in discussions about uncertainties whenever possible.

However, uncertainty is not solely detrimental; it can also foster motivation for problem-solving [18,32], stimulate curiosity [3,21,25,45], encourage information-seeking behavior [3,18,21,32,92], and recall [21]. Positive effects of uncertainty include increased openness in communication [45,58] and enhanced self-regulation, leading to greater resilience [3,21,23,25,58,66,73,78,112] and tolerance for uncertainty [32,58,66]. Moreover, uncertainty can drive veterinary medical research [12] and vigilance [18], contributing to advancements in the field.

## 4. Causes and Origins of Uncertainty Among Veterinary Professionals

The causes of uncertainty in veterinary professionals are complex and under-researched. Due to the random distribution of tolerance to uncertainty and its dynamic, individualized nature [41,53], it is likely impossible to create a comprehensive list of all potential causes and origins. Existing research often addresses only a limited subset of these causes, frequently examining them in isolation. Consequently, the related human medical literature can be confusing or overly simplistic in its categorization of uncertainty [3,5,12,19,27,39,113]. For example, while there is a recognized distinction between ambiguity and uncertainty, as well as uncertainty arising from clinical reasoning, there is a notable absence of discussion regarding system-related uncertainties. Furthermore, the categories presented are often limited, with most attempts at categorization in the medical field encompassing only a few aspects.

**Figure 3:**
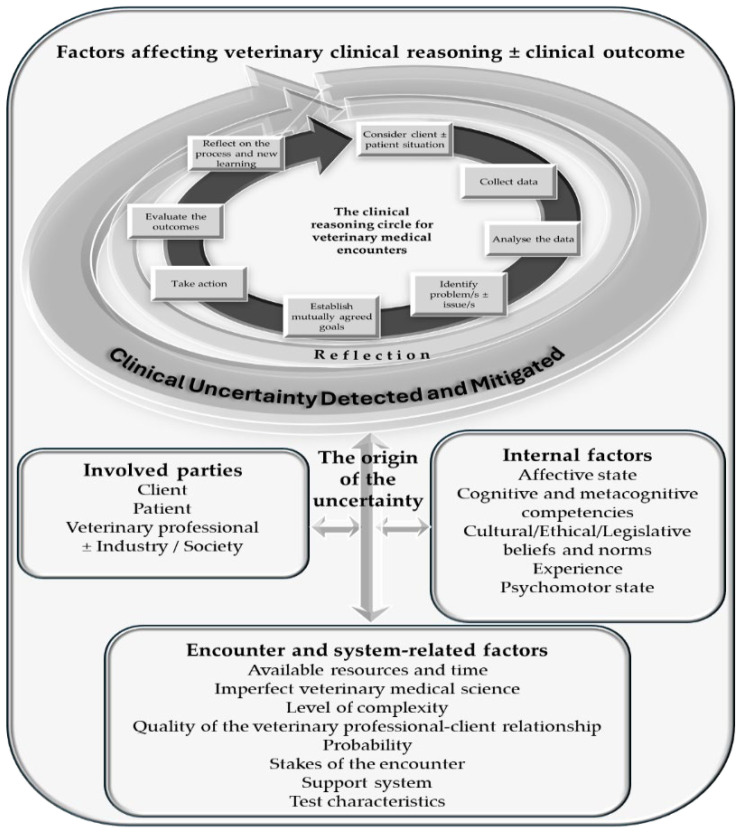
Intertwining of origins of uncertainty is common, and these may occur at any stage of the veterinary medical clinical reasoning cycle. For the best outcomes, uncertainty should be promptly detected and mitigated.

Many causes of uncertainty are interconnected, making it challenging to isolate any single factor [3,5,11] (Figure 3). Cognitive and socio-economic factors are often intertwined with emotional and behavioral aspects. Additionally, the origins of uncertainty frequently align with contexts related to clinical reasoning [114]. 

**Figure 4:**
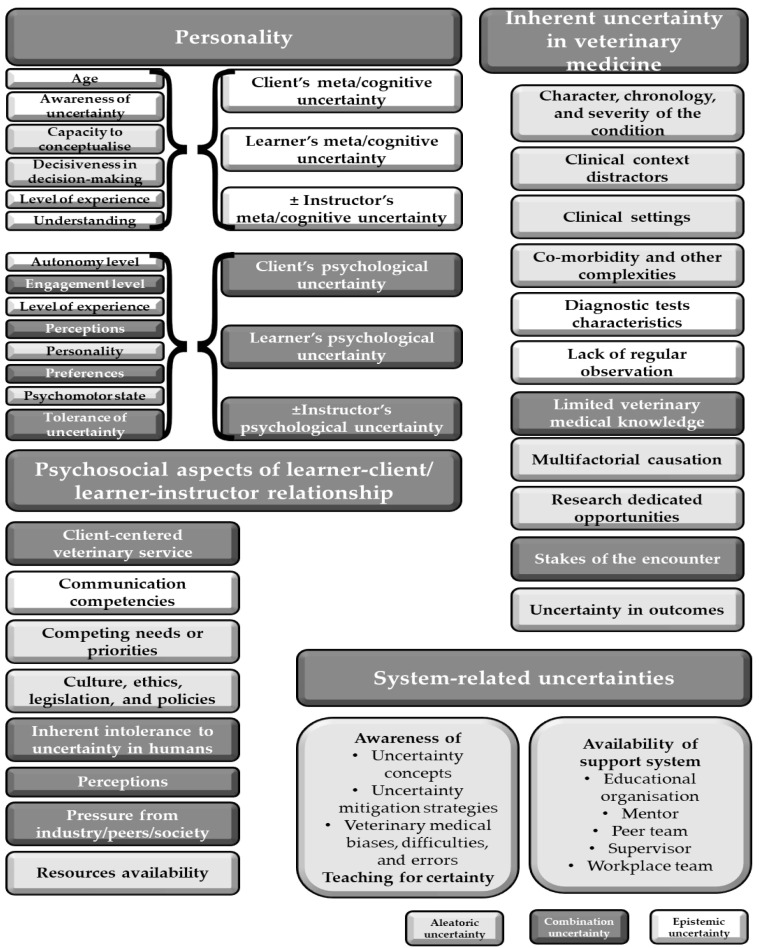
Common causes and origins of uncertainty in veterinary medical learners. White-filled rectangles indicate mainly aleatoric uncertainty. Light-gray-filled rectangles indicate mainly epistemic uncertainty. Dark gray-filled rectangles indicate a combination uncertainty.

Uncertainty can emerge at any stage of the clinical reasoning cycle, and both the veterinary professional and the client may experience concurrent uncertainties during an encounter. Some uncertainties are internalized; without effective communication, they may remain unexpressed and unresolved. We propose that the same or adjusted origins of uncertainty observed in human medicine are applicable to veterinary medical encounters. Figure 4 summarizes the origins of uncertainty identified in the medical literature [5,12,19,39,59], and although primarily focusing on veterinary medical learners, are also relevant to all veterinary professionals. Due to a significant lack of veterinary-specific literature on this matter, the authors have primarily derived information from medical literature.

The significant lack of comparative literature in both medical and veterinary fields regarding the three categories of uncertainty, namely aleatoric, epistemic, and their combination, complicates the ability to accurately assess the prevalence of each type. Consequently, the authors have developed a comparison between the two professions for each subcategory (Table 2) based on logical reasoning rather than evidence-based literature.

### 4.1. Inherent Uncertainty in Veterinary Medicine

#### 4.1.1. Uncertainties Related to the Nature of Veterinary Medicine

Many uncertainties in veterinary medicine stem from the inherent complexities of the field [1,3,6,9,12,13,17,19,20,21,22,25,26,30,32,39,43,46,48,74,79,82,84,92,96,104,106,115,116], including incomplete knowledge, the heterogeneity among individuals (such as variations in age), unpredictable changes in the likelihood of a patient being affected, and the overall complexity of veterinary care and science. The character, chronology, and severity of a presented condition can lead to atypical, inconsistent, or prominent signs and syndromes, as well as variable findings from ancillary techniques and tests [3,12,22,24,26,33,37,46,48,73,74,81,83,84,94,97,104,117,118].

For effective decision-making, it is crucial for veterinary professionals to observe and report signs and syndromes accurately. However, in extensive and large-scale intensive livestock production management, the observation of animals is often limited. As a result, important signs and syndromes may go unnoticed and unreported. Cognitive or mental impairments in clients can also contribute to the failure to report these signs and syndromes. Additionally, the perception of signs and syndromes can vary among different individuals, potentially compromising the quality of the information gathered. Clients who are less communicative may not report critical observations, further limiting the information available for veterinary decision-making.

This lack of comprehensive reporting can lead to incomplete access to crucial information [12], thereby increasing uncertainty during veterinary encounters. The lack of observation of animals is common in extensive and large-scale intensive livestock production management systems. Therefore, signs and syndromes may not be observed and thus, not reported [33,81]. Indeed, cognitive or mental impairment in the client may result in non-reported signs and syndromes [51,118]. Furthermore, signs and syndromes may be perceived differently by different people, limiting the quality of the obtained information [96]. Finally, signs and syndromes may not be reported by a client who is not verbose [96]. The complexity of the veterinary medical profession may be further complicated by comorbidities [27,33,96,104], concurrent involvement of various body systems [117], emergencies [21,29,38], and emerging or uncommon conditions [3,96]. Furthermore, many signs and syndromes are present in multiple conditions [3,12,48,51,74,81,97,117] and many conditions are multifactorial [12]. 

#### 4.1.2. Uncertainties Related to the Clinical Context

The clinical context plays a significant role in the prevalence of uncertainty among veterinary professionals [3,41,52,74,81,97,107]. Various distractors, often referred to as moderators [32], can influence decision-making in a manner similar to those affecting clinical reasoning. We have explored these factors in a previous article from our group [114].

One notable aspect of how clinical context impacts uncertainty is the stakes involved in a veterinary medical encounter. The potential consequences, whether for the client, the industry, the patient, or society, can heighten the level of uncertainty experienced [25,74]. For example, situations that pose risks to trade or have significant implications for animal welfare and the national economy can exacerbate feelings of uncertainty.

Further exploration of the psychosocial effects of the clinical context, particularly concerning the veterinary professionalclient relationship, is discussed in Section 4.3.

#### 4.1.3. Uncertainties Related to the Clinical Setting

Each clinical setting in veterinary medicine presents unique challenges and uncertainties [3,8,33,84,118,119,120]. For example, for veterinary learners, the classroom environment typically benefits from access to various teaching-assistance tools that may not be available in clinical practice [8,48,102,121]. However, despite these advantages, classroom learning lacks the dynamics and unpredictability inherent in real-world clinical situations. Consequently, uncertainties in traditional classroom settings are often more closely related to cognitive and metacognitive capacities.

In contrast, for all veterinary professionals, ambulatory fieldwork is associated with heightened uncertainties due to competing client needs, limited access to health records, and restricted access to diagnostic tests and management options [3,33,84,118]. While veterinary practitioners may prioritize immediate patient care, clients may be more focused on preventing future cases [116] or ensuring a consistent supply of high-quality products to the market, especially in production animal encounters. Fieldwork also presents challenges related to limited opportunities for literature research and various distractions, such as noise. As a result, uncertainties in this setting encompass not only cognitive and metacognitive factors but also compliance, diagnostic, management, and system-related uncertainties.

Hospitalized encounters introduce their own set of complexities, including a higher risk of nosocomial infections and the need for extensive care [84]. In these settings, uncertainties extend beyond cognitive and metacognitive issues to include diagnostic decisions, patient safety, and prognosis.

Specialist encounters add another layer of complexity, as they require veterinary professionals to meet the expectations set by primary veterinary practitioners. This dynamic can introduce additional uncertainties related to communication and collaboration among professionals.

#### 4.1.4. Uncertainties Related to Limited Veterinary Medical Knowledge

Limited veterinary knowledge significantly contributes to the prevalence of uncertainty among veterinary professionals, often referred to as epistemic uncertainty. Like all medical fields, veterinary learners or professionals are trained to rely on evidence-based veterinary medicine [12,24,27,28,39,43,46,56,84,85,106]. However, during their educational journey, they may develop a false sense of security regarding the efficacy of evidence-based approaches. Current curricula for veterinary learners can foster the belief that there is always a clear answer to clinical questions [81,122].

As learners and professionals transition into practice, the scarcity of evidence-based literature becomes apparent [51,123], leading to an increase in uncertainty. This transition often involves changes in the workplace, from primarily hospital environments to ambulatory or outpatient settings, which can further heighten uncertainty. In some cases, the limited cognitive and metacognitive resources of veterinary professionals may also contribute to this uncertainty [12,30,41,48,59,96,103]. Additionally, many areas of veterinary medicine lack established practice guidelines, which can leave practitioners without clear directions [3,21,33,37,41,48,51,116]. Even the best guidelines cannot completely eliminate uncertainties [81,122]. 

Some uncertainties arise from the imperfect characteristics of diagnostic tests [3,24,26,41,44,74,84], leading to conflicting findings in the literature [12,43]. This inconsistency complicates decision-making as veterinary professionals often must rely on their own, sometimes unreliable, experiences when evaluating management strategies or prognoses [3,12,19,25,33,44,46,59,80,85].

The exponential growth of veterinary medical knowledge, while beneficial, paradoxically increases complexity and amplifies uncertainty [19,101]. Most literature addressing the inherent causes of uncertainty originates from the medical field, with limited research focused specifically on veterinary medicine. Therefore, we urge veterinary researchers to investigate the inherent causes of uncertainty in veterinary encounters and to explore whether additional distinct sources of uncertainty exist.

### 4.2. Personality and Its Relationship to Uncertainty in Veterinary Medicine

No two individuals in the world will always make the same decision, and this variability is partly attributable to personality, often referred to as aleatoric uncertainty [3,31,39,53,85,87,118]. Different people may have varying perceptions of what is considered ‘normal’ or ‘abnormal’ in clinical contexts [12,32,43,74,96].

In an ideal scenario, the key performance indicators (KPIs) for any condition would serve as the gold standard for evaluation. However, these KPIs may not be acceptable to high achievers, nor may they be realistically achievable for clients at the lower end of the performance spectrum. This divergence in expectations and perceptions can contribute to uncertainty in decision-making among veterinary professionals.

#### Personality Traits Related to Variability in Tolerance of Uncertainty

Human nature exhibits a minimal tolerance for uncertainty and an inherent need for certainty [18,31]. Individuals show intrinsic variability in their tolerance to uncertainty [2,5,8,12,15,19,20,21,22,23,24,25,26,27,28,29,30,31,32,33,34,35,36], as well as in their learning capacities and preferences [46]. This variability in tolerance can change over time and be improved through educational approaches and repeated practice [35,98,121,122,124]. Research indicates that medical professionals with a higher tolerance for uncertainty consistently provide better client and patient care [2,31,36,125], actively seeking solutions and thriving in uncertain environments [46]. For veterinary professionals to effectively address cases, they must possess a belief in their ability to solve them, at least to some degree [11].

A lower tolerance for uncertainty in clinical reasoning and practice is a common source of stress and burnout among medical professionals [1,6,21,23,35,44,76,77,90,92,98,114]. It is reasonable to assume that this phenomenon also applies within the veterinary field. However, the literature on the factors influencing tolerance for uncertainty is limited and occasionally contradictory. The effects of uncertainty and the likelihood of its disclosure are influenced by numerous factors, as summarized in Table 3. Due to the significant lack of veterinary-specific information, the authors primarily derived their insights from human medical literature. The table was created by summarizing the findings from studies or reviews that often addressed multiple factors influencing tolerance.

Veterinary professionals may experience a higher prevalence of uncertainty when clients lack an understanding of the clinical encounter [19,24,84,96,97] (e.g., inability to participate in shared decision-making, or uncertainty regarding how a diagnostic test would improve animal welfare). This understanding is influenced not only by education but also by professional experience [3,9,10,15,23,25,37,38,44,62,85,87,98,106,125]; less experienced professionals in the medical field often report higher anxiety and find uncertainty less acceptable.

Individuals exhibit variations in their cognitive and metacognitive competencies [3,16,19]. Metacognitive skills, including conceptualization [3,13,41,44,53,92], decisiveness in decision-making, and problem-solving abilities, significantly impact comprehension. Drawing from medical literature, veterinary practitioners must adeptly apply their medical expertise across diverse clinical situations and species. To accomplish this, they need to be flexible in selecting and applying guidelines and proficient in transferring their competencies to different contexts.

Decisiveness in the decision-making process is influenced by both cognitive and metacognitive factors, which can delay or hinder a person’s ability to make decisions effectively. Cognitive elements include, but are not limited to, the lack of awareness of biases, difficulties (including uncertainty), and errors in clinical reasoning [16,17,37], or ineffective veterinary medical competencies, including communication [3,39,44,53,75,85]. Metacognitive elements include, but are not limited to, flexibility in clinical reasoning [37,87], the mental organization of knowledge [13,38,53], understanding clients’ perspectives [3,43,59] (e.g., recognizing the client’s viewpoint and how management strategies impact overall health and productivity).

Veterinary professionals often experience heightened uncertainty when clients lack understanding of the clinical encounter (e.g., inability to engage in shared decision-making or uncertainty regarding how a diagnostic test might enhance animal welfare). Comprehension is shaped not only by educational background but also by professional experience; for instance, less experienced practitioners in the medical field may exhibit increased anxiety and reduced tolerance for uncertainty. Understanding is also influenced by metacognitive competencies such as conceptualization, decisiveness in decision-making [25,87], and problem-solving skills [13]. Veterinary professionals should adeptly apply their medical knowledge across various clinical scenarios. This requires flexibility in guideline selection and the ability to transfer skills across diverse encounters and species.

### 4.3. Psychosocial Aspects of the Veterinary Professional–Client Relationship

The quality of the veterinary professional–client relationship significantly affects the prevalence of uncertainty [3,24,31,44,96]. Both clients and veterinary professionals possess competing needs and priorities [3,12,66,96,108,118], which can influence their interactions and, consequently, the level of uncertainty experienced. Clients must navigate relationships with veterinary professionals, other advisors, employees, and family members, which complicates their decision-making process.

The psychosocial states of both clients and veterinary practitioners are crucial for managing and tolerating uncertainty [52,66,125]. These states are influenced by individual circumstances and external factors such as societal pressures or global events like pandemics [66]. Personal circumstances may include, but are not limited to, attitudes, emotional states, cognitive overload, fatigue, multitasking, sleep deprivation, and stress [87,107,114].

#### 4.3.1. Uncertainties Related to Perceptions and Expectations

Outcomes expectations from the encounter may differ between the client and the veterinary professional [12,15,96]. This discrepancy can lead to uncertainty due to the client’s lack of knowledge regarding expectations or the presence of unrealistic expectations.

All individuals, including clients and veterinary professionals, perceive their environments uniquely [37,52]. Therefore, perceptions regarding the client and patient [29,115], preferences for veterinary care [3,37], and specific encounters may contribute to uncertainty. Many veterinary professionals believe that clients are intolerant of uncertainty and prefer clear, definitive management plans [3,21,32,47,56,104]. Common perceptions include clients exhibiting negative emotions [29,31,44,47,96,97] or distrust [15,39,44,47,53,96,115] when uncertainty is disclosed. While some studies indicate that clients react negatively to disclosed uncertainty, others find that clients appreciate such transparency [104,115]. Another common belief is that admitting uncertainty reflects a lack of competence [53,57]. Moreover, veterinary professionals often assume that clients from higher socio-economic backgrounds are more educated and more tolerant of uncertainty [97]. Lastly, a common misconception is that clients primarily seek financial advantages from outcomes.

#### 4.3.2. Uncertainties Related to Contextual Influences

Certain psychosocial aspects of the interaction and the veterinary professional–client relationship are components of the broader context, previously discussed under Section 4.1. Psychosocial factors affecting uncertainty prevalence include individual preferences [118], quality of the veterinary professionalclient relationship [97,115], personality traits [3,59,118], professional autonomy [7,21,53], and psychomotor state [3,38,45] (e.g., distress). For instance, medical, and likely veterinary, learners rely on team support during their education, but upon entering practice, they often face decisions alone [23,73]. This lack of a supportive team can be particularly pronounced in fieldwork or rural settings [79,118].

#### 4.3.3. Uncertainties Related to Communication Competencies

Communication is a critical factor influencing the quality of the veterinary professional–client relationship [54,55,94,96]. In client-centered veterinary services, it is essential to provide clients with opportunities to make informed decisions [37,96,104,115]. This is only achievable if all aspects of the encounter are discussed transparently and uncertainties are disclosed. Examples of effective communication strategies are included in the Supplementary Material.

### 4.4. System-Related Uncertainty in Veterinary Education and Practice

System-related uncertainty and its effects in veterinary education and practice may be associated with clinical settings, pressures affecting decision-making in clinical reasoning, and the presence or absence of a supportive team.

#### 4.4.1. Uncertainty Related to the Veterinary Clinical Settings

Each clinical setting presents unique uncertainties, as previously discussed under the heading ‘Inherent uncertainty in veterinary medicine’. Additional common system-related uncertainties pertain to the competing needs of the clients and veterinary professionals [3,10,108,118], and resource availability (e.g., limitations during fieldwork). Resource availability [3,19,21,23,43,118] relates to access to diagnostic techniques/tests, financial considerations, the use of software and artificial intelligence to assist in decision-making [23,37,130], and, for veterinary learners, teaching methodologies [121]. Interestingly, the integration of software and artificial intelligence can paradoxically introduce new uncertainties [3,27,43].

#### 4.4.2. Pressures Affecting Decision-Making in the Clinical Reasoning of Veterinary Practitioners

System-related uncertainties encompass various pressures impacting the veterinary encounter [3,19,21,118]. These pressures may arise from client expectations (e.g., demands for immediate resolutions), cultural factors [31,32] (e.g., inability to euthanize an animal that suffers), ethics [3,38,39,44,45,51] (e.g., client’s autonomy), industry [74] (e.g., limitations in available treatment options), and legislative requirements [47] (e.g., legal obligations to disclose risks associated with management options). Additionally, peer influence [10,19,21,32,41,52] and organizational policies [74] and societal norms [10,32] can shape perceptions of competence and urgency [3,19,21,26,51,74,121]. The time constraints become more evident in modern urban veterinary medical workplaces. In some cultures, disclosing uncertainty may be viewed as a failure [7,19]. Finally, organizational or workplace culture may discourage the disclosure of uncertainty [3,41,44,52]. 

#### 4.4.3. Effect of Support System on Uncertainty

The prevalence of system-related uncertainties is closely linked to the availability of adequate support systems [3,8,31,41,44,79,98], and awareness of biases, difficulties, and errors in clinical reasoning [16,40]. A lack of supportive culture and team dynamics typically results in increased uncertainty and potential long-term effects on veterinary professionals. The context of uncertainty disclosure, particularly if perceived as unsafe, can exacerbate feelings of uncertainty [45]. Additionally, the need for a support system that accommodates diverse learning preferences remains underexplored [128].

### 4.5. Teaching for Certainty

Uncertainty often arises from the misconception among veterinary professionals that only one optimal management approach is valid, whereas multiple management options may be appropriate [1,3,42,58,85]. Current veterinary education tends to create an illusion of certainty [7,19,26,32,42,58,81,83,98,101,115,122,131], often unintentionally (e.g., through assessment methods that reward “correct” answers). This emphasis on certainty can lead learners and practitioners to feel inadequate when faced with uncertainty [1,8,17,31,39,83,90,98,131]. Emerging veterinary professionals prioritize knowledge essential for clinical practice and requirements for assessments. Consequently, epistemic uncertainty may arise from teaching and assessment modalities [6,79,125,131], particularly those that emphasize absolute truth (e.g., True-False questions) or singular best answers (e.g., Multiple-Choice Questions). Such methods diminish tolerance for uncertainty [1,2,8,34,83,104,131] and hinder the development of metacognitive competencies like clinical reasoning and problem-solving [81,98,122]. While direct observation methods, such as Direct Observation of Procedural Skills (DOPS) or Objective Structured Clinical Examination (OSCE), are considered effective, they still lean towards a single-best-answer approach [2,81]. Medical, and likely veterinary learners who are educated in systems prioritizing certainty typically develop an aversion to uncertainty [8,42,83,131]. Additionally, concerns regarding litigation or other repercussions lead many assessment tasks to focus on singular correct answers, further discouraging the admission of uncertainty [8].

The dogma of certainty in veterinary medicine does not end with the completion of veterinary education. Certainty is also favored in veterinary clinical practice. The traditionalism of certainty is also visible in the diagnostic coding system and clinical decision tools [1,3], with a lack of ‘I don’t know’ diagnosis, meaning uncertainty in veterinary medicine is not acceptable.

## 5. Recognition of Struggles with Uncertainty

In an ideal scenario, veterinary professionals would seek assistance when confronted with medical uncertainty. However, in reality, many are reluctant to acknowledge their struggles (e.g., fear of failing a course, losing client opportunities, or experiencing status loss among peers) [27,31,41,46,53,62,105]. Supervisory teams should be equipped to recognize when veterinary professionals are grappling with undisclosed uncertainty. Literature addressing this issue is limited or vague [3,15,26,27,35,132]. For this purpose, a supervisory team may use the proxy indicators, including but not limited to those listed in Box 1. Due to a significant lack of veterinary-specific literature on this matter, the authors have primarily derived information from medical literature.

Box 1 **Indicators that a veterinary professional struggles with uncertainty.**

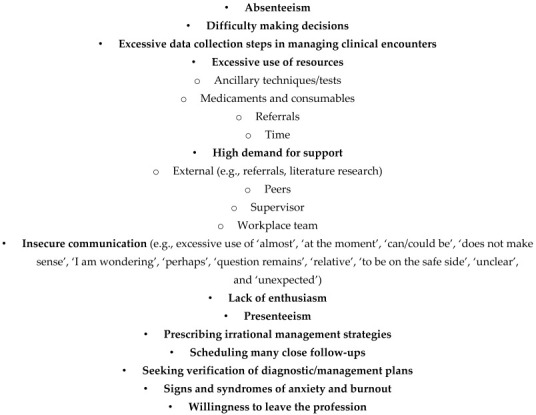

Recognition of a veterinary professional’s struggle with uncertainty is futile without timely intervention utilizing appropriate mitigation strategies. Such interventions should aim to prevent long-term negative outcomes, particularly concerning the mental health of the veterinary professional.

## 6. Mitigating Uncertainty

Mitigation is necessary primarily for harmful uncertainties [18]. No universal ‘one-size-fits-all’ solution for uncertainty exists. Mitigation strategies must be tailored to the specific type of uncertainty encountered. For example, uncertainty resulting from fatigue necessitates mindfulness and rest, while cognitive uncertainty may require cognitive restructuring or continuing education. The literature inadequately addresses the relationship between the root causes of uncertainty and corresponding mitigation strategies [58]. 

Mitigation should start with diagnosing the root cause of the uncertainty. As the root cause may be in several areas (Figure 4), it may not be easily diagnosed. Mitigation strategies overall should target two key areas: addressing problems and managing emotions [18]. Problem-focused mitigation strategies aim to resolve the root causes of uncertainty through cognitive or behavioral interventions [6]. In contrast, emotion-focused strategies aim to alleviate discomfort stemming from uncertainty. 

The science of uncertainty mitigation is still in its infancy, with many unknowns remaining. Notably, some cognitive and behavioral interventions have diminished the impact of problem-based learning (e.g., cognitive-forcing strategies related to medical knowledge, modifications in assessment, and simulations) but have inadvertently increased emotion-based uncertainties [6]. Consequently, focusing solely on the technical aspects of the curriculum may be counterproductive; each mitigation strategy should address all three dimensions of uncertainty: behavioral, cognitive, and emotional. Research into effective uncertainty mitigation strategies is warranted.

Mitigation of uncertainty, as a metacognitive competency, can be partially learned. An awareness of mitigation strategies is a good start. Yet, veterinary professionals should be aware that even the best continuing and self-directed education will fail to provide them with all possible answers. Therefore, accepting some level of uncertainty tolerance in veterinary medical encounters is necessary.

**Figure 5:**
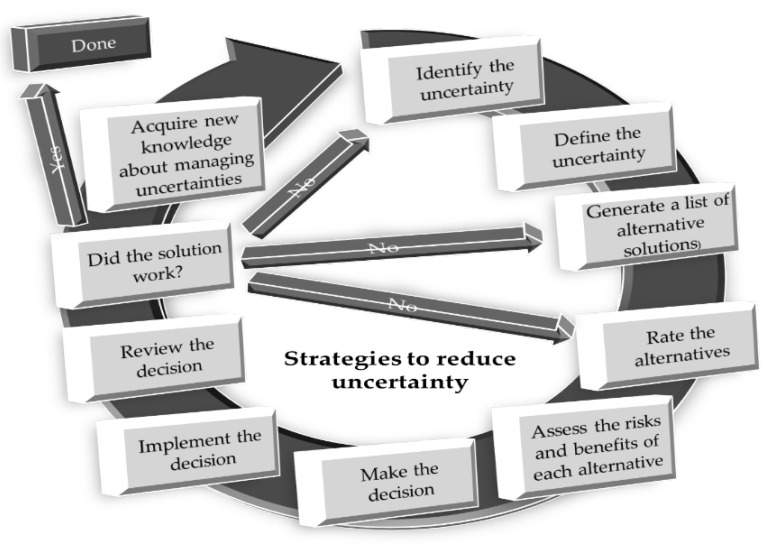
Mitigation of uncertainty using the modified decision-making process applicable to veterinary medical problems, in this case, uncertainty [133]. In addressing uncertainty in veterinary medical issues, a modified decision-making process can be employed. Initially, the uncertainty must be identified. For effective mitigation, it is crucial to define the specific nature of the uncertainty. Each identified uncertainty can be mitigated through multiple strategies, which should be listed and prioritized based on their potential effectiveness. The next step involves assessing the potential risks and benefits of these strategies, ultimately leading to a decision on the most appropriate mitigation strategy or strategies. Once a strategy is selected, it should be implemented and the outcomes must be reviewed. If the chosen mitigation strategy proves effective, the process is considered to be nearing completion. To finalize the cycle, veterinary practitioners should utilize the insights gained from this process to develop new knowledge applicable to managing uncertainties in future situations. However, if the selected mitigation strategy does not alleviate the uncertainty, the process must be repeated, as this may indicate that the original uncertainty was not accurately identified or defined.

Several learning/teaching uncertainty mitigation strategies are available in human medicine, and a limited number in veterinary medicine literature, with many being empirical only. Furthermore, even for strategies with evidence-based literature, many reports are contradictory. In our opinion, learning/teaching uncertainty mitigation strategies should include those listed in Table 4. Our approach was to register proposed strategies dependent on the root cause of uncertainty (Figure 5).

Additional material on mitigation is provided in the Supplementary Material, using a case study example. We would point out that in clinical practice, as many of the uncertainties are multifactorial, concurrent use of multiple mitigation strategies will be required. The use of multiple mitigation strategies has been shown to have a positive impact in addressing uncertainty [6,40,96].

**Table 4:** Proposed mitigation strategies for a variety of causes of veterinary medical uncertainty. Note that some uncertainties are difficult to mitigate (noted as NA—not applicable).

Origin of Uncertainty	Factor	Proposed Strategies	References
Inherent uncertainty in veterinary medicine	Character, chronology, and severity of the condition	An assessment methodology that enables tolerance of variability in the presentation of morbidity	[2,6]
		Cognitive forcing strategies; Collecting sufficient (but not excessive) data; Continuing education	[1,3,6,10,13,18,21,44,48,58,74,134]
		Debrief	[34]
		Dedicated research opportunities	[3,18,74]
		Developing clinical reasoning competency of the veterinary professional	[10,48,79]
		Developing general and clinical problem-solving skills	[6,10,11,13,95]
		Peer discussions/teaching	[3,8,10,34,45,58,74]
		Repeated practice	[19,34,134]
		Use of artificial intelligence	[130]
		Use of clinical teaching models (e.g., The five microskills, SNAPPS)	[1,24,28,48,83,95,135,136,137]
		Use of other clinical teaching strategies (e.g., diagnostic pause, role modeling)	[1,27,28]
	Clinical encounter context distractors	Address the distractors	[41,74]
		Vigilance	[18]
	Clinical settings	Ensuring good orientation and setting expectations	NA
	Comorbidity and other complexities	An assessment methodology that enables tolerance of comorbidity/other complexity	[2,6]
		Cognitive forcing strategies; Collecting sufficient (but not excessive) data; Continuing education; Repeated practice	[1,3,6,18,21,44,48,58,102]
		Dedicated research opportunities	[18,58,74]
		Gamification	[6,95]
		Peer discussions/teaching	[58,102]
		Use of artificial intelligence	[130]
	Diagnostic tests characteristics	Awareness of diagnostic test limitations	[1,48,56,96,108,132]
		Consider the use of an alternative diagnostic test	[74]
		Peer discussions/teaching	[58]
		Repeated practice	[10,48,74,95]
		Use of artificial intelligence	[130]
		Use of evidence-based veterinary medicine	[48,58,67,74,87]
		Use of technology, including artificial intelligence	[74]
	High-stake encounter	Awareness of high-stakes encounters	[75,96,128]
		Awareness of expected communication	[75]
		Cognitive forcing strategies	[48,75,128]
		Developing general and clinical problem-solving skills	[6,13,133]
		Peer discussions/teaching	[128]
		Regular use of reflective practice	[6,34,74]
		Repeated practice	[75,96]
	Lack of regular observation	Client education	NA
		Regular analysis of the client’s records to stimulate record-keeping	NA
	Limited veterinary medical knowledge	Assisting the mental organization of knowledge	[13]
		Cognitive forcing strategies	[13,18,48,58]
		Facilitated discussion intertwined with uncertainty	[1,3,8,22,23,24,28,38,95]
		More research; Preparation of veterinary medical practice guidelines	[3,48,51,58,116]
		Peer discussions/teaching	[34,58]
		Use of artificial intelligence	[130]
	Multifactorial causation	An assessment methodology that enables tolerance of multifactorial causation	[2,6]
		Cognitive forcing strategies; Collecting sufficient (but not excessive) data; Continuing education	[1,3,6,18,21,44,48,58]
		Dedicated research opportunities	[18,58,74]
		Regular use of reflective practice	[34,84]
		Peer discussions/teaching	[34,58]
		Repeated practice	[21,34,48]
	Research dedicated opportunities	Allowing time for research related to uncertainty	[58,74]
	Uncertainty in outcomes	Cognitive forcing strategies	[48]
		Peer discussions/teaching	[34,58]
		Regular use of reflective practice	[3,6,32,34,42,74,84,95]
		Use of artificial intelligence	[130]
		Use of clinical teaching models (e.g., The five microskills, SNAPPS)	[1,24,28,83,95,136,137]
Personality	Age	NA	NA
	Autonomy level	Gradual development of professional autonomy in veterinary professionals	[53]
		Repeated practice	[6,16,34]
	Awareness of uncertainty	An assessment methodology that enables tolerance of uncertainty	[2,6,8,34,102]
		Cognitive forcing strategies	[8,40,101,102]
		Facilitated discussion intertwined with uncertainty	[1,3,6,8,22,23,24,28,38,83,95]
		Gamification (e.g., tactical decision games)	[6,95,121]
		Inclusion of uncertainty in veterinary medical education curricula and continuing education events; Tolerating uncertainty as a ‘normal’ occurrence in veterinary medicine	[3,6,8,17,21,22,27,32,40,42,43,44,48,87,106]
		Peer/Workplace team members’ discussions	[1,3,17,22,45,102]
		Reflective practice	[8,48,84,102,122]
		Simple verbal acknowledgement of uncertainty by instructors	[8,84,102,122]
		Understanding that, despite all professional development, some level of uncertainty will be a regular occurrence in veterinary clinical practice	[21,22,37,83]
		Use of clinical teaching models (e.g., The five microskills, SNAPPS)	[8,48,122,137]
	Linguistic imperfections	Effective communication; Use of summary and clarification	[12,54,55,104]
	The client’s cognitive uncertainty	Assess the uncertainty in the client and discuss it further	[19,54,55]
		Include elements of shared decision-making	[44,48,84,93]
		Use of a safety net approach	[3,13,19,59,84,93,96,104]
	Client’s psychological uncertainty	Assess the uncertainty in the client and discuss it further	[19,54,55]
		Provide emotional support to the client	[19,54]
	Capacity to conceptualize	Developing general and clinical problem-solving skills	[6,13,76,133]
		Developing clinical reasoning competency of the veterinary professional	[31,79,86]
		Repeated practice	[21,42,44]
	Decisiveness in decision-making	Developing general and clinical problem-solving skills	[13,76,133]
		Developing clinical reasoning competency of the veterinary professional	[79]
		Utilize artificial intelligence and technology	[17,27,42,43,48,117]
	Engagement level	Use of clinical teaching models (e.g., The five microskills, SNAPPS)	[1,24,28,136]
		Use of other clinical teaching strategies (e.g., diagnostic pause, role modeling)	[1,27,28]
	Prevention of bias	Awareness of common biases	[16,40,81]
		Use of artificial intelligence	[130]
	Veterinary professionals’ cognitive uncertainty	Cognitive forcing strategies; Collecting sufficient (but not excessive) data; Continuing education; Repeated practice	[1,3,6,17,19,21,24,44,48,85]
		Facilitate the development of cognitive and meta-cognitive competencies	
		Facilitating a transfer between the types of clinical reasoning; Opting more towards the analytical type of clinical reasoning	[21,27,43,78,79]
		Facilitating reflective practice	[81]
		Improving the organization of veterinary medical knowledge in a clinically relevant manner	[6,37,44]
		Peer/Workplace team members’ discussions/teaching	[1,3,10,17,22,58,62,121]
		Use of a safety net approach	[3,19,42,84,87,96,104,138]
		Use of clinical teaching models (e.g., The five microskills, SNAPPS)	[1,24,28,136,137]
		Use of other clinical teaching strategies (e.g., diagnostic pause, role modeling)	[1,27,28]
		Vigilance	[18]
	Veterinary professionals’ psychological uncertainty	Adjusting attention	[58]
		Adjusting epistemic expectations	[58]
		An assessment methodology that enables tolerance of uncertainty	[2,6]
		Gradual development of professional autonomy in veterinary professionals	[53,87]
		Exercising flexibility	[58]
		Include elements of shared decision-making	[22,44,78,84,90,93]
		Participate in work–life balance activities (e.g., arts, social functions, sports)	[36,76,112]
		Prioritizing uncertainty	[58]
		Regular use of debriefing for veterinary medical learners and early-career veterinary professionals	[1,6,48,121,136]
		Repeated practice	[42,48]
		Regular use of reflective practice	[1,17,22,32,34,48,74]
		Use of clinical teaching models (e.g., The five microskills, SNAPPS)	[1,24,28,136]
	Level of experience	Gradual development of professional autonomy in veterinary professionals	[53,87]
		Repeated practice	[21,31,44]
	Perceptions	Assess the assumptions	
	Preferences	Assess the preferences of the client for the encounter	[54]
	Psychomotor state	Exercising compartmentalization	[58]
		Regular use of debriefing for veterinary medical learners and early-career veterinary professionals	[1,3,6,53,79,121,136]
		Self-management strategies (e.g., improved work–life balance, relaxation techniques, and sports)	[23,36,58,76,112,123]
	Tolerance of uncertainty	Accepting that some tolerance of uncertainty is inevitable	[3,86,93,102]
		An assessment methodology that enables tolerance of uncertainty	[2,6,48,102]
		Inclusion of uncertainty in veterinary medical education curricula and continuing education events; Tolerating uncertainty as a ‘normal’ occurrence in veterinary medicine	[6,32,48,84,102]
		Disclosing and discussing the uncertainty increases the level of tolerance	[21,44,58,84,102]
		Participate in work–life balance activities (e.g., sports)	[36,76,112]
	Understanding	Development of effective veterinary professional–client relationship; Effective communication;	[29,42,54,55]
		Repeated practice	[31]
Psychosocial aspects of the veterinary professional-client relationship	Client-centered veterinary service	Identifying the client’s agenda, including the ‘hidden agenda’	[54,116]
		Include elements of shared decision-making	[22,44,78,84,90,93,102]
	Communication competencies	Assessing communication preferences by the client	[19,96,100,116]
		An assessment methodology that enables communication of uncertainty	[2,6,102]
		Effective communication both about and within situations of uncertainty	[3,19,22,24,27,28,44,53,54,55,56,58,116,136]
		Use of a framework for effective communication in clinical encounters	[42,54,55,100,116]
		Repeated practice	[54,55,102]
	Competing needs or priorities	Assessing the possibility of the presence of competing needs	[10,108,116]
	Culture, ethics, legislation, and policies	Familiarity with local and client culture; Familiarity with applicable ethics, legislation, and policies	[31,34]
		Repeated practice	
	Inherent intolerance to uncertainty in humans	Cognitive forcing strategies facilitate awareness and acceptance of tolerance	[1,3,6,98]
		Participate in work–life balance activities (e.g., sports)	[36,76,112]
	Perceptions	Assess the assumptions	[3,17,34,39,44,90]
	Pressure from industry/peers/society	Assess the causes of pressure	[116]
		Familiarity with industry requirements	
	Resources availability	Identification of available resources	
System-related uncertainty	Availability of organizational support system	Regular use of debriefing for veterinary medical learners and early-career veterinary professionals	[1,3,6,41,45,53,79,84,121,136]
	Availability of mentor/peer team/supervisor/workplace team	Debriefing; Discussions; Support	[1,3,6,21,32,34,45,53,58,62,74,121]
		Safe learning/working environment	[3,31,45,74,77]
	Awareness of concepts of uncertainty	Inclusion of uncertainty in veterinary medical education curricula and continuing education events; Tolerating uncertainty as a ‘normal’ occurrence in veterinary medicine	[3,6,17,19,21,22,27,28,43,44,56,67,75,85,90,92,102,106,136]
		Facilitate disclosure of uncertainty by creating a safe learning/working environment	[21,58,74,83,84,98,114,129]
		Peer discussions/teaching	[8,67,120,123,139]
		Peer/Workplace team members’ discussions	[1,3,17,22,84,121]
	Awareness of uncertainty mitigation strategies	Inclusion of uncertainty mitigation strategies in veterinary medical education curricula and continuing education events	[3,67,84,90,92,129]
		Peer/Workplace team members’ discussions	[1,3,17,22,98,103]
	Awareness of veterinary medical biases, difficulties, and errors	Inclusion of biases, difficulties, and errors in clinical reasoning in veterinary medical education curricula and continuing education events	[1,16,44,81]
		Use of a framework for remediating difficulties and errors in clinical reasoning	[16]
	Decreasing the workload	Use of artificial intelligence	[130]
	Teaching for uncertainty	An assessment methodology that enables tolerance of uncertainty and its communication	[2,6,34,129]
		Anonymous discussion forums	[34]
		Cognitive forcing strategies (e.g., role modeling, ‘think-aloud’)	[84]
		Facilitated discussion intertwined with uncertainty	[1,3,22,23,24,28,38,75,83,84,95,98,121]
		Gamification	[6,95,121]
		Reflective practice with informal feedback	[34,84,90]
		Repeated practice	[6,90,93,95,138]
		Teaching veterinary medicine humanities competencies	[6,8,31,34,95,102,140]
		Use of artificial intelligence	[130]
		Use of clinical teaching models (e.g., The five microskills, SNAPPS)	[1,24,28,83,95,136,137]

### 6.1. Teaching for Uncertainty

Awareness of uncertainty and mitigation strategies is essential and emphasized by accreditation bodies [63,64,65], but are currently inadequately addressed in veterinary medical curricula and ongoing professional education. In alignment with the principle that “prevention is better than cure” (Benjamin Franklin, 1736), a proactive approach to uncertainty may decrease mental health issues, including self-harm and suicidal tendencies, among veterinary practitioners.

Veterinary professionals must recognize that managing clinical encounters is rarely binary [1,19,81,85,122,126,131] and that many cases involve an inherent level of uncertainty [2,17,24,43,85,90,98,136]. Educational approaches should aim to enhance tolerance for uncertainty [40,57,67,98,102,105,124]. Therefore, fostering an acceptance of uncertainty should be integrated into the entire veterinary medical curriculum [48,67,120], starting in the pre-clinical years when learners are predominantly trained for certainty. Research advocates for early curricular intervention [6,17,34,44,90,102,116,127,134]. Teaching coping strategies for uncertainty can be structured as a separate course or embedded throughout the veterinary medical curriculum [17,32,43]. Frameworks similar to those developed for medical education should be applied [48]. Future efforts should focus on creating a framework specific to veterinary education, but incorporating uncertainty management into veterinary curricula does not eliminate the need for ongoing self-directed education.

#### 6.1.1. Shifting Assessment Paradigms

The emphasis in veterinary education should shift from a binary (certainty) perspective to recognizing uncertainty as an integral aspect of life and veterinary practice [2,19,22,26,48,74,81,89,106,122]. An initial step toward integrating uncertainty into veterinary education involves modifying assessment formats. This shift is particularly crucial for Generation Z and subsequent learners, who often prioritize outcomes over learning [127]. Introducing assessment questions with multiple correct answers, rather than solely relying on multiple-choice questions, represents a progressive move in this direction [26,43,48,56,81,102,106,127,131]. This approach encourages deeper learning and promotes acceptance of uncertainty. 

Alternative assessment methodologies, such as prioritization questions, where learners rank responses by likelihood [2,129,131], concept mapping [129], script concordance test [81,131], and short essay questions [131], should also be explored. However, logistical challenges and costs may hinder the implementation of these assessment strategies. Furthermore, reliance on single-best-answer assessments can create a false sense of security among learners, particularly those from Generation Z, ultimately diminishing their tolerance for uncertainty [127]. 

Decreasing the incentive of the assessment outcome, moving towards a fail or pass rather than a point system, is another step in the right direction [34,127]. A better stimulus for learning, rather than an assessment outcome, may be achieved by the utilization of programmatic assessment [141,142] (e.g., the (O)RIME system [129]), intertwined with regular feedback [4,142,143,144].

#### 6.1.2. Integration of Uncertainty Paradigms

Addressing uncertainty within veterinary education extends beyond assessment. Uncertainty paradigms should be integrated into existing curricula, encompassing both traditional and clinical teaching modalities [121]. Clinical teaching must facilitate the recognition and admission of uncertainty, enhancing confidence in its communication [90]. The sSupplementary Material provides a case study that illustrates this approach using the Five Microskills model of clinical teaching. 

#### 6.1.3. Instructor Preparedness for Teaching Uncertainty

Veterinary instructors are expected to teach about uncertainty; however, instructors in medical education have reported feeling unprepared for such responsibilities [95,101,120,121]. Unprepared instructors may inadvertently minimize or eliminate uncertainty in their teaching, resulting in an unrealistic educational experience [101,120,121]. We expect the same to apply to veterinary instructors. Therefore, it is essential for veterinary schools to adequately prepare instructors for teaching and assessing uncertainty.

### 6.2. Role of the Debrief in Dealing with Uncertainty

Utilizing debriefing as a metacognitive competency fosters deep learning and the development of metacognitive capacities in veterinary professionals [2]. Expressions of curiosity, doubt, and the pursuit of clarification enhance learning and competency development [135,136]. Debriefing should encourage connections between clinical presentations and pathophysiological mechanisms, thereby facilitating clinical reasoning competencies, including evaluation and synthesis [1]. Furthermore, debriefing provides an opportunity to alleviate uncertainty, as discussions with peers, instructors, and learning teams can mitigate stress [3,53,74,79,102,121]. Effective and constructive feedback is crucial, and guidelines for providing such feedback have been prepared by our team [143]. 

### 6.3. Organizational Culture and Support Systems

Organizations, such as universities and workplaces, must establish a supportive culture that fosters the expression of uncertainties. For organizations primarily involved in developing veterinary professionals, we recommend regular case conferences, ideally every two weeks, led by less experienced colleagues. This approach facilitates metacognitive competency development and enhances confidence [74]. All discussions on cases, performance, and uncertainties should occur in a safe and supportive environment. 

### 6.4. Reflective Practice

Effective reflective practice should encompass three elements: (a) preparing for uncertainty (reflection-for-action) [1,34,74], which enables planning for potential uncertainties and developing contingency plans, especially in high-risk scenarios (e.g., “What can be done if I am wrong?”); (b) addressing uncertainty as it arises (reflection-in-action) [74], which facilitates real-time problem-solving (e.g., “What can I do to decrease the confusing finding of XX?”); and (c) learning from past uncertainties (reflection-on-action) [1,34,84], enabling practitioners to implement preventive measures for future encounters (e.g., “Now that I know this is a potential uncertainty, what can I do to prevent it from happening again in future encounters?” or “How can I build resilience to prevent negative effects of the unknown in future encounters?”).

### 6.5. Effective Communication Strategies

A critical mitigation strategy involves effective communication through carefully crafted disclosures of uncertainty [29,48,62,92,100,145]. Such communication should foster trust rather than unnecessary anxiety within the veterinary professional-client relationship [1,3,19,42,44,47,104,115]. Understanding clients’ communication preferences allows practitioners to tailor discussions effectively (e.g., employing visual aids). Many medical professionals, including veterinarians, report feeling uncomfortable and unprepared to communicate uncertainty [29,62,86,90,94,100,104,108]. Therefore, our Supplementary Material [146,147] provides examples of communication strategies for addressing uncertainty, illustrated through a case study.

### 6.6. Limitations of Ancillary Tests

Discussing ambiguous findings (e.g., false positive laboratory results due to imperfect test sensitivity and specificity [1,132]) is crucial. Both clients and veterinary professionals should recognize that reliance on ancillary techniques and tests for diagnosis is misguided. An increased volume of tests and findings heightens the risk of false positives [56]. Clinical reasoning should remain rooted in comprehensive data collection, including health interviews and clinical examinations. Ancillary techniques and tests should serve as supportive tools rather than sole decision-makers. Furthermore, it is unrealistic to expect veterinary professionals to possess in-depth knowledge of all tests’ performance characteristics [132].

### 6.7. Role of the Admission Process in Mitigating Uncertainty

Research indicates that the admission process in veterinary schools may influence candidates’ tolerance for and preparedness to address uncertainty [11,33,35,76]. For example, candidates with sports backgrounds often demonstrate higher tolerance and better coping mechanisms for uncertainty [76]. Likewise, candidates entering medical or surgical programs through alternative pathways have shown greater readiness to handle uncertainty and life challenges [11]. Consequently, veterinary schools aiming to produce graduates with enhanced uncertainty tolerance should consider reevaluating their admission processes. However, caution is necessary to evaluate the broader impacts on workforce dynamics and trainee supply. A comprehensive assessment will help ensure that changes support the long-term sustainability and effectiveness of postgraduate training programs.

## 7. Gaps in Veterinary Literature and Translational Challenges Using Medical Literature to Apply Principles to the Veterinary Medical Field

Veterinary literature related to uncertainty is very limited. Some aspects of the methodology for the effects of illness and uncertainty [18], measuring uncertainty [33], reasons for mental health aspects in the profession [66,109,110,111,148], reasons for uncertainty [10], and tolerance of uncertainty [149], and how to teach it [10] are covered, but there is a significant lack of literature for informed decisions to be made. Uncertainty is mentioned in all major accreditation guides [63,64,65], yet evidence-based approaches to its understanding and mitigation are lacking.

As previously noted, this review primarily draws upon existing medical literature. We endeavored to paraphrase the principles pertaining to uncertainty without rigorously synthesizing their application to veterinary practice. For instance, epistemic uncertainty in veterinary education and practice is further exacerbated by the diversity of species, each possessing distinct morphological and physiological characteristics [150,151]. However, the limited literature on this subject reveals only a marginal difference in the tolerance for uncertainty between medical and veterinary learners [149]. Consequently, we acknowledge that the current scarcity of evidence-based literature within the veterinary medical field may yield definitive and potentially divergent conclusions in future research.

## 8. Conclusions

This review identified significant uncertainty-related gaps in the veterinary medical education literature. This review highlights the pervasive nature of medical uncertainty in veterinary practice, underscoring its impact on clinical reasoning, decision-making, and the veterinary professional-client relationship. By categorizing uncertainty into aleatoric and epistemic types, we elucidate the multifaceted origins of uncertainty that challenge practitioners. Despite its negative implications, uncertainty can also drive curiosity and foster problem-solving skills. We have emphasized practitioners, but recognize the need to address uncertainty throughout the career path, including education. Veterinary education must incorporate a robust framework for addressing uncertainty, promoting tolerance, and enhancing metacognitive competencies among professionals. Future efforts should focus on developing comprehensive training programs that equip veterinarians to navigate uncertainty effectively, ultimately improving the quality of care provided to patients and fostering stronger client relationships. Continuous exploration and adaptation of educational strategies are essential to cultivate a workforce capable of thriving amidst the complexities of veterinary medicine.

## Figures and Tables

**Table 1:** Types of impact resulting from uncertainty in veterinary medical education and practice, deduced from information combining medical and veterinary literature.

Type of Impact	Common Subtypes	Reference/s
Aversive cognitive manifestations	Cognitive discomfort and lack of confidence	[21,22,25,33,41,44,77]
	Decreased clinical reasoning competence	[15,17,21,26,78,79,80,81]
	Decreased tendency to adopt new techniques and technologies	[3,14,15,23,81]
	Interruptions in routine	[18]
	Tendency of decreased adherence to best practice guidelines	[82]
	Tendency to take shortcuts	[83]
Aversive emotional response	Avoidance of confrontation	[21,84]
	Carrier and job choice/dissatisfaction	[3,8,21,22,23,27,35,46,50,73,78,79,80,85,86,87,88,89]
	Decreased engagement (with the encounter, socially, or with the team)	[3,13,21,23]
	Decreased tolerance to uncertainty	[33,41,90]
	Maladaptive coping strategies	[1,3,9,18,21,23,24,27,32,35,44,45,73,85]
	Mental health and psychological well-being effects	[1,3,6,8,9,11,15,18,21,23,24,25,27,28,32,33,34,35,42,43,44,46,48,49,50,53,73,74,76,77,78,79,80,81,82,83,84,85,86,88,89,91,92,93,94,95,96,97,98,99,100,101,102]
	Professional fragility	[15,18,21,31,35,78,93,103]
	Reticence to recognize and disclose uncertainty	[13,15,19,58,62,74,77,80,81,92,93,94,98,101,103,104,105]
	Seeking a single best answer (certainty)	[2,19,22,26,98,106]
Limited professional development opportunities	Limited career and job choices	[3,21,22,23,27,33,73,78,82,85,96,101]
	Limited leadership opportunities	[3,46,98]
Suboptimal veterinary service provision	Altered patient safety	[2,13,15,18,21,31,37,62,76,79,86,90,98]
	Change in clinical practice approaches	[81,88]
	Client dissatisfaction	[96,97,100,101]
	Compromised veterinary professional-client relationship	[1,3,8,13,15,18,19,22,27,50,56,81,87,94,95,97,101,104]
	Decreased communication competency	[2,15,19,21,29,44,77,97]
	Decreased likelihood of shared decision-making	[2,19,21,37,44,81,84]
	Delayed decision-making	[8,11,21,22,26,37,39,41,58,87,96,103,107]
	Higher risk of introduction of biases and errors in clinical reasoning	[15,46,80,81,87,93,94,103,108]
	Ineffective use of resources	[1,2,3,9,13,15,17,22,24,25,26,31,33,41,43,48,49,56,58,59,73,74,76,77,78,81,82,83,84,85,86,87,88,90,92,93,94,96,98,104,105,108]
	Risk to trade	
	Suboptimal management	[11,37,103]
	Suboptimal care	[34,42,46,48,58,95,96]
	Unnecessary animal suffering	[15]
Positive effects	Ability to disclose uncertainty in future encounters	[95]
	Decreased level of errors in clinical reasoning	[8]
	Facilitation of problem-solving competencies	[8,21,25,32,87,92]
	Facilitation of open discussion	[45,82,95]
	Facilitation of veterinary medical research	[12]
	Greater client satisfaction	[8]
	Humility	[45]
	Increased resilience	[21,25,58,80]
	Openness to new ideas	[82]

**Table 2:** A summarized comparison of the prevalence of uncertainty between veterinary medical and human medical learners and practitioners.

Category	Subcategory	Comparison to the Uncertainty in Human Medical Fields
Aleatoric	Age	Probably the same
	Awareness of uncertainty	Probably the same
	Client’s meta/cognitive uncertainty	Probably the same
	Communication competencies	Probably the same
	Diagnostic test characteristics	Probably the same, although medical practitioners have better access to regular updates
	Lack of regular observation	Probably higher, as animals are not closely observed, particularly in extensive production systems
	Instructor’s metacognitive uncertainty	Probably the same
	Learner’s meta/cognitive uncertainty	Probably the same
	Level of experience	Probably the same
	Understanding	Probably the same
Epistemic	Availability of the support system by the educational organization	Probably the same
	Availability of the support system by a mentor	Probably the same
	Availability of the support system by a peer team	Probably the same
	Availability of the support system by information technology, including software	Probably higher, as fewer technologies, including software, are available to veterinary practitioners
	Availability of the support system by a supervisor	Probably the same
	Availability of the support system by the workplace team	Probably the same
	Awareness of uncertainty concepts	Probably the same
	Awareness of uncertainty mitigation strategies	Probably the same
	Awareness of veterinary medical biases, difficulties and errors	Probably the same, although these are less presented in veterinary medical education
	Capacity to conceptualize	Probably the same
	Character of the morbidity	Probably higher, as there is less evidence-based literature available to veterinary practitioners
	Clinical context distractors	Probably higher, particularly in ambulatory practice, as often there is a lack of appropriate facilities to prevent several distractors
	Clinical settings	Probably higher, particularly in ambulatory practice, as often there is a lack of appropriate facilities
	Comorbidity	Probably higher, as there is less evidence-based literature available to veterinary practitioners
	Competing needs or priorities	Probably the same
	Culture	Probably the same
	Decisiveness in decision-making	Probably the same
	Ethics	Probably the same
	Legislation	Probably the same, although some legislation is restrictive to the options for management available to veterinary practitioners
	Multifactorial causation	Probably higher, as there is less evidence-based literature available to veterinary practitioners
	Personality	Probably the same
	Policies	Probably the same, although some policies are restrictive to the options for management available to veterinary practitioners
	Psychomotor state	Probably the same
	Resources availability	Probably higher, as there are less equipment, evidence-based literature facilities, and guidelines available to veterinary practitioners
	Teaching for certainty	Probably the same, or higher, as medical education has already included uncertainty in many curricula
	Uncertainty in outcomes	Probably the same, although medical practitioners have higher access to regular updates
Combination	Client-centered veterinary services	Probably the same
	Client’s psychological uncertainty	Probably the same
	Engagement level	Probably the same
	Inherent intolerance to uncertainty in humans	Probably the same
	Instructor’s psychological uncertainty	Probably the same
	Learner’s psychological uncertainty	Probably the same
	Limited veterinary medical knowledge	Probably higher, as there is less evidence-based literature available to veterinary practitioners
	Perceptions	Probably the same
	Preferences	Probably the same
	Pressure from industry/peers/society	Probably the same
	Stakes of the encounter	Probably lower (as medical professionals deal with human life; except when the veterinary encounter has potential public health implications)

**Table 3:** Factors affecting tolerance of uncertainty and their effect on the likelihood of its disclosure by veterinary professionals, based primarily on human medical literature. Strength of the evidence: - No evidence; + Low; ++ Medium; +++ High.

Parameter	Characteristic	Effect on Uncertainty Tolerance			References
		Negative	Neutral	Positive	
Age	Increase	++	+	++	[8,11,27,36,57,98,126]
Awareness of uncertainty	Presence	-	+	+++	[5,17,21,26,32,83,87,90,96,98,125]
Belief in certainty in medicine	Presence	++	-	-	[1,2,32,104]
Burnout	Presence	+++	-	-	[36]
Cognitive/Metacognitive capacity	Higher	-	+	++	[3,51,90,102]
Complexity of the encounter	Complex	+	-	-	[3,97]
Country of practice	Developed	-	-	+	[36]
Country of training	Developed	-	-	++	[36,126]
Cultural background	Prohibitive	+++	-	-	[8,17,23,31,32,51,97,108]
Discussion of uncertainty with the client	Common	+	+	++	[36,57,97]
Educational background and knowledge of the client	Higher	+	+	++	[5,88,97]
Ethnicity	Prohibitive	++	+		[36]
Gender	Female	+++	++	++	[11,27,37,57,82,89,98,107,126]
Generation characteristics	Z and above	+	-	-	[127]
High resilience	Presence	-	-	++	[36]
Intrinsic tolerance of uncertainty	Present	-	+	++	[8,31,36,57,88,98,107]
Lack of veterinary medical knowledge	Present	++	+	-	[48,96,128]
Length of professional experience	Longer	-	+	+++	[8,13,15,23,27,31,32,34,45,46,51,77,83,87,89,90,92,93,98,107,124,125,126]
Length of workplace learning placement	Longer	-	-	+	[93]
Linguistic imperfections	Present	++	+	-	[12,39,51,97]
Organizational culture	Prohibitive	+++	-	-	[8,17,44,45,51]
Organizational structure	Highly hierarchical	+++	-	-	[17,27,44,45,97,103]
Participation in extracurricular activities (e.g., sports)	Regular	-	-	+	[76]
Peer discussion of uncertainty	Common	-	-	+	[97,103]
Perception of ethical or moral issues	Presence	++	+	-	[3,17,43,104]
Perception of risk of being seen as incompetent	Presence	++	+	-	[1,11,27,31,36,44,94,96,98,103,129]
Perception of risk of malpractice	Presence	+++	-	++	[19,34,36,44,57,82,87,88,90,98]
Perception of risk of repercussion	Presence	+++	-	-	[8,27,31,32,82]
Perception of the lack of evidence	Present	++	-	-	[36,39,48]
Perception of unwanted economic effects	Present	+	++	-	[36]
Personal beliefs, views and values	Against	+++	-	-	[15,31,36,39]
Preparedness to disclose uncertainty	Presence	-	-	++	[8,27]
Prior history of similar experience	Presence	++	+	-	[11,23,26,44,87,96]
Psychosocial state	Altered	+++	-	-	[8,15,23,27,36,45,66,87,98,107]
Sociodemographic factors	Prohibitive	++	+	-	[36,126]
Supportive team	Presence	-	-	+++	[3,17,31,32,34,41,44,57,66,93,97]
The stakes of the encounter	High	++	+	+	[1,27,31,57,97]
Traumatic events (e.g., pandemics)	Presence	+	-	-	[66]
Veterinary field of work	Emergency or Surgery	++	-	++	[3,13,15,29,36,57,59,75,80,82,89,90,97,98,105,108]
Work–life balance	Good	-	-	+	[36]

## Data Availability

No new data were created or analyzed in this study.

## Supplementary Materials

The following supporting information can be downloaded at: https://www.mdpi.com/article/10.3390/vetsci12121203/s1, Supplementary Material: Dealing with Clinical Reasoning Uncertainty in Veterinary Medical Encounters with a Clinical Example.

## Glossary

**Term**	**Meaning**
Aleatoric (uncertainty)	Uncertainty arises from the fundamental nature of reality and sampling selection being unpredictable. Synonyms: metaphysical or stochastic uncertainty.
Ambiguity	The perception of uncertainty in diagnosis, treatment, or prognosis for a clinical encounter due to the presence of information that is vague, unclear, or open to multiple interpretations (epistemic uncertainty).
Analytical type of clinical reasoning	Based on more deliberate, explicit, purposeful, rational, and slow, and focuses on hypothesis generation and deductive reasoning that is closer to the cognitive processes associated with problem-solving. Common synonyms: Deductive, Deliberate, Rational, Rule-governed or System/Type 2 clinical reasoning.
Clinical encounter context distractors	Situational factors arising from the patient, physician, or environment that draw attention away from the primary clinical objectives and can negatively affect the clinical reasoning process and diagnostic accuracy.
Clinical reasoning	The cognitive process interjected with unconscious operations during which a learner or practitioner collects information (clinical and context), processes it, comes to an understanding of the problem presented during a clinical encounter, and prepares a management plan, followed by evaluation of the outcome and self-reflection. Common synonyms: Clinical/Diagnostic/Medical: Acumen/Cognition/Critical thinking/Decision-making/Information processing/Judgment/Problem solving/Rationale/Reasoning.
Clinical teaching	A form of interpersonal communication between a clinical instructor and a learner that involves a physical or virtual clinical encounter.
Cognition	A mental activity or a process of acquiring knowledge and understanding.
Cognitive forcing strategies	A group of interventions that use mechanisms to disrupt the heuristic processing of information. It is part of the metacognitive approach
Combination uncertainty	The overall uncertainty in an encounter that arises from the joint presence of both aleatoric and epistemic uncertainty. Common synonyms: mixed, predictive, and total uncertainty.
Context (in clinical reasoning and veterinary encounters)	A complex interaction of factors (including but not limited to affective/physical state, client, encounter, environment, finances, patient, and social environment) that affects the clinical reasoning competence of the learner/veterinary professional.
Deep learning	Learner aims for mastery of essential academic content; thinking critically and solving complex problems; working collaboratively and communicating effectively; having an academic mindset; and being empowered through self-directed learning.
DOPS (Direct Observation of Procedural Skills)	Workplace-based assessment tool, used to evaluate a learner’s performance of a procedure.
Dual type of clinical reasoning	Clinical reasoning that utilizes concurrently the analytical and intuitive types. Common synonyms: Dual-/Mixed–process clinical reasoning/theory.
(Clinical/Veterinary) encounter	Any physical or virtual contact with a veterinary patient and client (e.g., owner, employee of an enterprise) with a primary responsibility to carry out clinical assessment or activity.
Epistemic (uncertainty)	Uncertainty that arises from the lack of knowledge, scientific evidence, or understanding of the issue. Synonyms: cognitive or uncertainty of ignorance (medical ambiguity). NOTE: This uncertainty, theoretically, can be lowered with more data and research.
(Clinical/Veterinary) instructor	A person who, in addition to the regular veterinary practitioner’s duties, is a clinical instructor should fulfill the roles of assessor, facilitator, mentor, preceptor, role model, supervisor, and teacher of veterinary learners in a clinical teaching environment. It may include any of the following: Apprentice/intern in the upper years, Resident, Veterinary educator/teacher, or Veterinary practitioner.
High-stake encounter	Clinical encounters associated with a high-stress environment or situation (e.g., euthanasia, loss of a patient, or population-level emergency)
Intuitive type of clinical reasoning	Based more on cognitive shortcuts (e.g., heuristics) than real intuitive (Gestalt effect) processes. Therefore, even the intuitive type of clinical reasoning is not equal to the real meaning of intuitive (‘judgment made quickly and without apparent effort’). Common synonyms: Experiential, ‘Gut feeling’, Inductive, Non-analytical, Tacit, or System/Type 1 clinical reasoning.
(Veterinary medical) learner	A person studying to become a veterinarian, doctor or surgeon of veterinary medicine and/or surgery, or a veterinary paraprofessional, encompassing individuals from vocational students to those in advanced veterinary medical programs. Common synonyms: apprentice, student.
Metacognition	Critical awareness of one’s thought processes and learning, and an understanding of the patterns of thinking and learning (‘thinking about thinking’).
Nosocomial	Originating in a hospital or other animal health facility, and was not present at the time of admission.
OSCE (Objective Structured Clinical Examination)	Assessment methodology that typically consists of a circuit of multiple exam stations, each with a different task, which learners visit rotating
Psychosocial causes of medical uncertainty	Uncertainties that stem from the interaction between an individual’s psychological state and their social environment, creating ambiguity or a lack of sureness in healthcare situations.
Reflection	The metacognitive process that may occur before, during or after an encounter aims to develop a deeper understanding of the encounter and self ± the team to inform the ongoing and/or future actions, behaviors, and encounters.
Reflection-for-action	A process of self-evaluation of the action to happen, including planning for action and performing the action, anticipating the unexpected, and planning and executing adjustments from before, during and after the encounter.
Reflection-in-action	A process of self-evaluation of the action as it happens, resulting in ongoing adjustments during the encounter.
Reflection-on-action	A process of self-evaluation of the action after it has been completed, planning for adjustment in future encounters.
Safety net approach	A strategy used in medical and veterinary encounters to manage diagnostic uncertainty and ensure client and patient safety by providing information on potential risks and unknowns, empowering the client to actively participate in the management.
SCT (Script Concordance Test)	Learners’ assessment methodology based on the extent to which learners’ responses on a clinical case reflect those of an ‘expert’ panel.
Self-management	Taking responsibility for one’s own well-being and achievement of personal or professional goals, by controlling own actions, behaviors, emotions, and thoughts.
SNAPPS model of clinical teaching	A learner-centered model of clinical teaching: 1. Summarize briefly the history and findings; 2. Narrow the differential to two or three relevant possibilities; 3. Analyze the differential by comparing and contrasting the possibilities; 4. Probe the preceptor by asking questions about uncertainties, difficulties, or alternative approaches; 5. Plan management for the patient’s medical issues; and 6. Select a case-related issue for self-directed learning.
The Five Microskills model of clinical teaching	An instructor-centered model of clinical teaching: 1) Get a commitment; 2) Probe for supporting evidence; 3) Teach general rules; 4) Reinforce what was done well; and 5) Correct mistakes. An additional stage is the ‘Debrief’.
Tolerance of uncertainty	The ability of a person to accept that things are unpredictable, to cope with the complex/unfamiliar/unknown situation, and their behavioral, cognitive, and emotional response.
Uncertainty	The dynamic, subjective perception of an inability to provide an accurate explanation of the patient’s health problem or the client’s worries resulting in behavioral, cognitive, and emotional responses.
Workplace (learning)	An educational method that immerses the learners in the workplace. Common synonyms: Experiential learning; Exposure to practice.
