# Supplementary material for: Clinical Reasoning Uncertainty in Veterinary Medical Encounters with a Clinical Example
Source: Vet Sci. 2025 Dec 15;12(12):1203. doi: 10.3390/vetsci12121203 (PMC12737725; doi:10.3390/vetsci12121203)
Supplement: Supplementary file 1 [file vetsci-12-01203-s001.zip › vetsci-4009906-supplementary.pdf]

Supplementary material

Supplementary Material: Dealing with Clinical Reasoning Uncertainty in Veterinary Medical Encounters with a Clinical Example

Roy Neville Kirkwood [1] and Kiro Risto Petrovski [1,2,*]

1 School of Animal and Veterinary Sciences, The University of Adelaide, Roseworthy, SA 5371, Australia; roy.kirkwood@adelaide.edu.au
2 Davies Livestock Research Centre, School of Animal and Veterinary Sciences, The University of Adelaide, Roseworthy, SA 5371, Australia; kiro.petrovski@adelaide.edu.au
* Correspondence: kiro.petrovski@adelaide.edu.au

This supplementary material discusses the recognition and management of clinical reasoning uncertainty within veterinary medical encounters, exemplified through a case study of sudden death in dairy cows. It emphasizes the necessity for veterinary professionals to develop skills in acknowledging and addressing uncertainty, as current guidance is limited. The authors adapt the Five Microskills model of clinical teaching and the eight stages of the clinical reasoning cycle to enhance veterinary education. The case presented involves an experienced dairy farmer whose cows exhibit sudden deaths without prior warning, prompting an investigation that includes post-mortem examinations and data collection.

The material outlines specific stages of the clinical reasoning cycle, detailing the process of data collection, analysis, and problem identification, leading to the formulation of a management plan. The case emphasizes the multifactorial nature of the veterinary medical encounter, highlighting uncertainties related to etiology, chronology, and management options. The authors advocate for open discussions about uncertainties in clinical practice, fostering a "safe learning environment" where both learners and instructors can engage in reflective practice.

Furthermore, effective communication strategies are proposed to facilitate conversations about uncertainty with clients and team members, underscoring the importance of transparency and shared decision-making. The material concludes with a glossary of key terms and concepts relevant to clinical teaching and reflective practices in veterinary medicine, aiming to equip veterinary professionals with the necessary tools to navigate uncertainty effectively in their practice.

Citation: Kirkwood, R.N.; Petrovski, K.R. Dealing with Clinical Reasoning Uncertainty in Veterinary Medical Encounters with a Clinical Example. *Vet. Sci.* **2025**, *12*, x. https://doi.org/10.3390/xxxxx

Academic Editor(s): Patrick Butaye

Received: no date
Accepted: no date
Published: date

Publisher's Note: MDPI stays neutral with regard to jurisdictional claims in published maps and institutional affiliations.

Copyright: © 2025 by the authors. Submitted for possible open access publication under the terms and conditions of the Creative Commons Attribution (CC BY) license (https://creativecommons.org/licenses/by/4.0/).

1. Introduction

The ability to recognize and address uncertainty is a critical competency for veterinary professionals (e.g., [1-3]). However, there remains a notable lack of guidance on how to foster the recognition and acceptance of uncertainty among veterinary medical learners, their educators, and practicing professionals. This document aims to establish preliminary guidelines for teaching the acknowledgment and management of veterinary uncertainty, utilizing a case example as a framework.

Our team has previously adapted the the Five Microskills Model of Clinical Teaching to enhance veterinary educational practices (for further details, see [4]; Fig. 1). Additionally, we have explored the resolution of veterinary medical encounters through the eight stages of the clinical reasoning cycle, which often involves iterative movement between these stages (for additional information on the clinical reasoning cycle, see [5]; Fig. 2). It is important to note that uncertainty may arise throughout the clinical reasoning process, which does not necessarily follow a linear trajectory [6]. While the Five Microskills Model has been recognized as effective for addressing learners' uncertainties [7], only the initial three steps have been adapted for clinical reasoning contexts thus far. This appendix will employ all steps of the Five Microskills Model to illustrate the introduction of veterinary medical uncertainty in both teaching and practice.

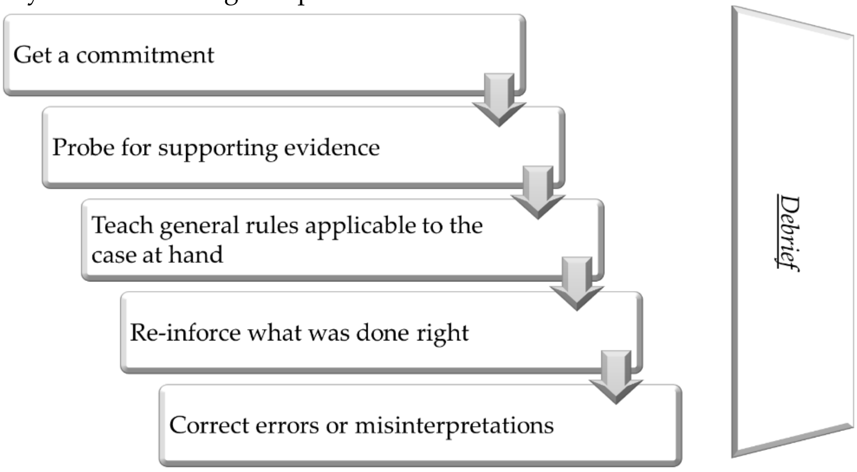

Figure 1. Steps of the Five microskills model of clinical teaching in veterinary medical encounters. Produced using information from [4].

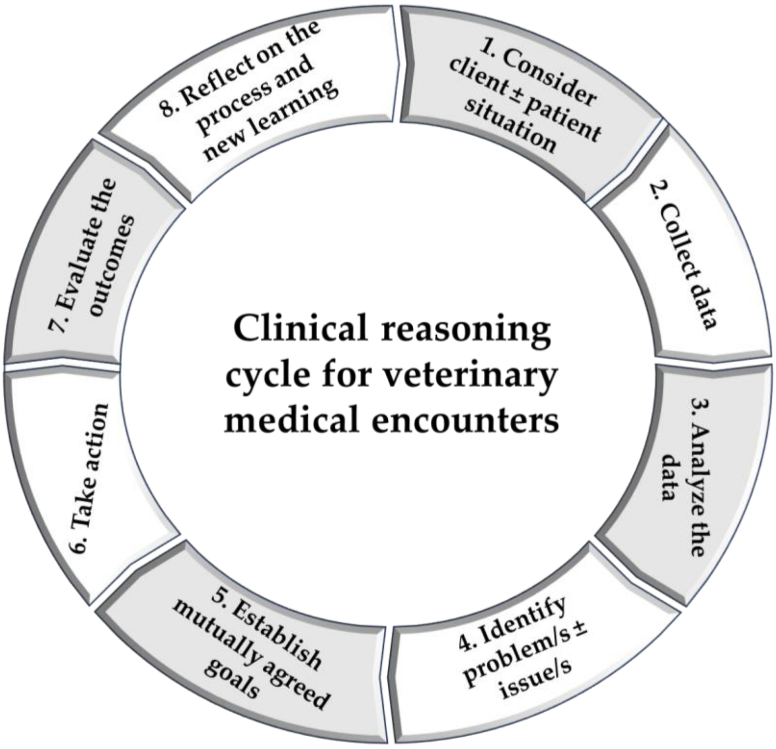

Figure 2. Clinical reasoning cycle for dealing with veterinary medical encounters. Produced using information from [5,8].

2. Example case

We propose that veterinary medical learners should approach each clinical encounter using the eight stages of the veterinary clinical reasoning cycle. This approach often requires simultaneous engagement with multiple stages or oscillation between them. The following example illustrates a real clinical case involving sudden deaths in dairy cows within a pasture-based, seasonal-calving system (see Table 1). In this scenario, the role of the learner and instructor is to assist the local veterinary practitioner, who may lack experience in dairy cattle medicine. Additionally, a livestock insurance company is involved in the investigation. Sudden deaths are a relatively common occurrence across various animal species, making this case suitable for teaching clinical reasoning and handling uncertainties. In the table below and elsewhere, italicized text means verbal communication (to differentiate from Latin terms, which are underlined).

Table 1. Stages of the veterinary medical clinical reasoning cycle, using an example of a cow presented as 'not well'. Note that italicized text means verbal communication.

Stage	Activity / Element	Example of a veterinary medical learner's synthesis of information
1. Consider the client ± patient situation	NA	The case concerns Mr. John Doe, an experienced dairy farmer, who reports the sudden deaths of 30 lactating dairy cows over a four-day period, with no prior warning signs. All deceased cows were in excellent condition and high producers.
2. Collect data	Presenting problem	A case of 30 lactating dairy cows dying over the past 4 days, with the death occurring without premonitory signs.
	Health interview	The farm ranks in the top 10% for milk production and quality nationally. The current herd comprises approximately 850 milking cows and 150 dry stock, including 80 calves, 68 heifers, and 2 bulls, all of which are purebred Holstein Friesians. The cows are milked twice daily through a rotary milking system with 64 units, with the last calving having occurred three weeks ago and the breeding season commencing one week ago.<br>The entire herd has been grazing on perennial ryegrass pastures, and all fatalities have occurred following the introduction of renovated paddocks approximately two weeks prior. Due to significant flooding four months earlier, a larger portion of the paddocks was renovated this year, resulting in increased reliance on stored feed. The diet consists of pasture (50%–70% of dry matter requirements), palm kernel extract (up to 3 kg per cow per day), grass silage (20%–30% of dry matter), and washed potatoes (10%–15% of dry matter). Mineral supplements from a reputable supplier are regularly provided (2% of dry matter). Nitrogen fertilizer was applied to all paddocks six weeks ago, and effluent is regularly sprayed in paddocks near the milking shed. Over the past two weeks, the weather has fluctuated, characterized by sunny but cool and drying conditions.<br>The farm is situated outside known anthrax regions, and there have been no significant changes in infrastructure or management practices over the past year. Vaccination against clostridial diseases and leptospirosis is irregular, primarily occurring in calves.<br>Mr. Doe contacted his usual veterinarian on the first day of the sudden deaths, who has since been attending the farm daily to conduct post-mortem examinations. A total of seven post-mortem examinations have been performed, and relevant reports are available. The livestock insurance company is also involved in the investigation.
	Various examination steps	**Environmental and Husbandry Observations:** No abnormalities were detected (NAD) in the environment or husbandry practices. The property features flat terrain with a river bisecting the land, and no noxious agents, such as rubbish dumps, sources of stray voltage, or toxic plants, were identified.<br>**Clinical Examination Findings:** None; the cows died without premonitory signs. Examination findings from 15 randomly selected cows in good condition revealed no abnormalities. Samples collected included blood for hematological and biochemical analysis, rumen fluid, and urine.<br>**Post-Mortem Examination Findings:** Three relatively fresh carcasses were available for examination. The post-mortem revealed significant findings, including the absence of soil disturbance around the carcasses, rapid post-mortem autolysis, notable bloating, and clotted blood emanating from the natural orifices (especially the nostrils, with less from the anus and vagina). Frothy fluid was observed in the nostrils of two out of three carcasses.<br>Upon opening the carcasses, no evidence of a bloat line or abnormal rumen contents was found. Findings included petechial hemorrhages in the kidneys and omentum, severe proliferative and hemorrhagic abomasitis, and empty small intestines in one carcass. The liver showed changes indicative of acute to subacute liver fluke in one of the carcasses.<br>**Samples collected:** Abomasum, Liver, and Kidney (submitted); Feces (tested on-site); Bone, Brain, Fat, and Muscle (stored)
	Ancillary examination techniques/tests	Further examinations yielded no significant findings in blood biochemistry or hematology performed with a portable analyzer, fecal analysis, urinalysis via a veterinary dipstick, or rumen content tests.<br>Culturing samples from the abomasum, liver, and kidney revealed an abundant growth of *Paraclostridium sordellii*.<br>Other tests conducted showed no additional significant findings, and tissue histology was not performed.
3. Analyze the data and 4. Identify problem/s ± Issue/s	Review data / Problem representation	Mr Doe is an experienced dairy farmer who has presented a case of 30 lactating dairy cows in good body condition and high levels of milk production dying over the past 4 days, with the death occurring without premonitory signs in cows in early lactation. On post-mortem examination severe hemorrhagic and proliferative abomasitis were detected in septicemic carcasses that rapidly autolyze. Culture yielded heavy growth of *P. sordellii.*
	Review context	Mr Doe is frustrated and stressed. The client is concerned about animal welfare and sustainability, as this is his family's livelihood.
	Problem identification	Sudden death in 3.5% of HF cows in high body condition and high milk yield.<br>Irregular vaccination (not since calves) against clostridial diseases and leptospirosis.<br>Indications of soil disturbance (ie. flooding and pasture renovation).<br>Indications of soil intake (ie. diet including washed potatoes and soil disturbance).<br>Indications of stress (ie. early lactation, high production).<br>No premonitory signs before death.<br>Out of the known anthrax area.<br>Rapid decomposition in carcasses.<br>Clotted blood ± foamy discharge coming out of natural orifices.<br>Indications of septicemia (ie. petechial hemorrhages in kidneys and omasum).<br>Severe hemorrhagic and proliferative abomasitis.<br>Heavy growth of *P. sordellii.*
	Recall knowledge	The initial working hypothesis is clostridial abomasitis, supported by the heavy growth of *P. sordellii.*

Stage	Activity / Element	Example of a veterinary medical learner's synthesis of information
	Interpretation	The rapid post-mortem decomposition and the presence of clotted blood suggest a clostridial disorder. The finding of the blood-tinged, swollen and inflamed abomasum, coupled with the external carcass signs, indicates a specific form of clostridial disease referred to as clostridial abomasitis.
	Discrimination	The absence of poisonous plants or dangerous goods within the paddocks helps to rule out poisoning or electrocution as potential causes.
	Relating	Ingested soil, a common habitat for clostridial bacteria, likely contributed to the increased presence of these pathogens. Given the dietary components and the management practices observed, clostridial abomasitis is strongly suspected as the primary etiology.
	Inferring	Washed potatoes may still have some soil on them. Additionally, the flooding and pasture renovation have resulted in soil disturbance. All these factors together have led to the ingestion of soil by cows.
	Matching	Clostridial bacteria grow well in the guts of cows fed on high-energy diets.
	Predicting	*Based on the information you provided and the findings of the necropsy, we strongly suspect clostridial abomasitis. Therefore, to stop further deaths immediately, we strongly recommend vaccination with a clostridial vaccine that contains ten types of clostridial bacteria. Additionally, in about two to three days, we should receive laboratory results confirming or rejecting the diagnosis.*
5. Establish mutually agreed goals	NA	Mr. Doe's primary concern is to halt the deaths, which have resulted in significant economic losses and anxiety regarding the welfare of his livestock. While vaccination immunity may take up to one week to develop, it is crucial to inform the client that additional deaths may still occur during this period.<br>Given the suspected bacterial etiology and the sensitivity of clostridial bacteria to penicillins, systemic antimicrobial administration may be warranted for high-risk cows over the next three to four days. Concurrent administration of antimicrobials with the toxoid vaccine is permissible. Additionally, the energy density of the cows' diet may need to be reduced until the vaccine achieves full efficacy.<br>Risk factors must also be addressed, considering both the economic implications and the feasibility of proposed management strategies. The economic justification and feasibility of the potential strategies should be accounted for.
6. Take action	NA	Although the primary cause of the sudden death syndrome appears singular, the epidemiology of suspected clostridial abomasitis is multifaceted. Recognized risk factors include early lactation stress, high-energy diets, high production levels, soil ingestion, and irregular immunization practices.<br>Immunization against clostridial diseases is a cost-effective preventive strategy; however, the delay in immunity development necessitates consideration of additional management measures to prevent further losses. The "take action" phase of the clinical reasoning cycle may involve employing a metaphylactic approach to manage the clostridial disease, focusing on identifying high-risk cows.<br>Adjusting the diet by reducing the amount of washed potatoes could yield two benefits: lowering the diet's energy density and decreasing soil ingestion. However, such changes may lead to reduced production and an increased risk of negative energy balance, potentially affecting reproductive performance. Moreover, decreasing pasture intake may not be feasible, and increasing silage proportions could similarly affect energy density.
7. Evaluate the outcome	NA	Management of the sudden death syndrome should be effective shortly after vaccination (approximately one week later) or more rapidly if a metaphylactic approach is employed (potentially within a day).
8. Reflection and new learning	NA	Comprehension of the etiology, epidemiology, pathophysiology, post-mortem findings, and diagnostic tests associated with the sudden death syndrome should facilitate reflective learning for the trainee. The health interview data, combined with environmental and post-mortem examination findings, should enhance real-time reflective practice for the learner. The selection of samples collected and tested should also encourage reflection and serve as a foundation for new learning topics for veterinary practitioners.

3. Practical application of the Five microskills model of clinical teaching using the case example

Numerous uncertainties may arise related to this case study. Common uncertainties are outlined in Box 1.

Box 1. Veterinary medical uncertainties that are commonly related to the example case
Multifactorial etiology of the syndrome
Chronology of the problem
Common risk factors
Sequence of occurrence of the conditions
Selection of the preferred management option
Stakes of the encounter (for Mr. Jahn Doe)

In this encounter, it is important to set the stage for recognition and acceptance of uncertainties (normalize the occurrence of uncertainty) [9]. For instance, the instructor may state, "*Clinical practice often entails navigating multiple possibilities, which complicates establishing a definitive diagnosis.*" or "*Those who seek a clear yes or no answer may not grasp the complexities of our field*" or "*We all feel uncertain at some moments. Experiencing uncertainty is a shared human and professional experience and acknowledging it can ease our discussions.*". Such comments can foster open discussions about uncertainty [7]. Recognizing that conversations around uncertainty can be stressful for learners, it is vital to cultivate a "safe learning environment." Discussions may need to be conducted away from the client or reserved for debriefing sessions.

The subsequent discussion will focus on uncertainties specific to the case example, without delving into the Five Microskills Model in detail. Readers are encouraged to refer to our previous publication for further exploration [4].

3.1. Get a commitment

When uncertainty arises in a learner's presentation, the instructor should actively promote open dialogue. For instance, the instructor could ask, "*Can we discuss the level of*

certainty regarding the potential causes of the sudden death syndrome observed in this case?" By expressing their own uncertainties, instructors can create an environment conducive to discussion. For example, an instructor might say, "*At this moment, I am uncertain about how long it will take for the vaccine to become effective.*"

3.2. Probe for supportive evidence

Expressing uncertainty provides a valuable opportunity to encourage self-reflection among learners [7]. To facilitate this reflection, instructors can pose questions such as:
a. "*What aspects of the sudden death syndrome make you feel more or less certain?*"
b. "*How would you determine the likelihood of lead toxicosis as a diagnosis, and what steps would you take to confirm it?*"
c. "*What are the common causes of sudden death, and are there distinguishing features among them?*"
d. "*Which diagnoses related to sudden death should never be overlooked, even if they seem unlikely?*"

3.3. Teach general rules

Instructors should model effective strategies for managing uncertainty [7,10]. For example, they might say, "*In cases of uncertainty, it is crucial to weigh the pros and cons while involving the client in the decision-making process.*" Instead of merely stating general rules, instructors can share their thought processes [7]. They might explain, "*In this scenario, it is likely that the sudden death syndrome is linked to soil ingestion, either from pasture or washed potatoes. However, identifying the primary source is challenging due to the widespread nature of the causative pathogen.*"

3.4. Reinforce what was done well

Acknowledging uncertainty can facilitate better management of it [7,11]. As part of effective feedback, instructors should recognize and validate learners' acknowledgment of uncertainty. For example, "*Well done for recognizing that the sudden death syndrome in this case is intertwined with nutritional risk factors. It is commendable that we all admit uncertainty regarding the primary etiology at this moment.*"

3.5. Correct mistakes

Learners may often hesitate to recognize or admit uncertainty. Therefore, effective feedback should encourage this recognition. For instance, an instructor might say, "*We can all benefit from acknowledging that, at this time, we cannot predict the outcome of the immunization.*"

3.6. Debrief

Debriefing is a vital component of reflective practice that is essential for addressing uncertainty within veterinary education and practice [4,10,12-20]. Instructors should stimulate reflection among learners for all encounters, especially those associated with uncertainties. For example, "*What is your understanding of the diagnostic uncertainties related to the sudden death syndrome, and how can this knowledge be applied to similar cases?*"

The debriefing session should encompass not only the veterinary medical aspects of the case but also the non-medical and psychosocial dimensions. For example, "*Can we discuss the emotional impact on the client when faced with the realities of this uncertain situation?*"

Communication competency

Effective communication is characterized by attentive listening [6,21,22], and assertiveness. Linguistic inaccuracies, such as the use of technical jargon, can significantly contribute to client uncertainty [6,22,23]. As part of the uncertainty mitigation, communication competency is usually related to communication with the client (Tables 2 and 3).

Communication competency is vital for fostering a strong veterinary professional-client relationship [20,23-26] and is critical for successful shared decision-making [9,12,19,20,23,24,27-31]. Shared information should allow for the client's autonomy and informed decision-making [30-33].

There is a scarcity of frameworks addressing the effective communication of uncertainty in medical fields [6,22,29,34,35]. We have previously outlined practical and theoretical foundations for effective communication in veterinary encounters [21,28]. However, discussions on uncertainty have been notably sparse in these works. In the subsequent tables (Tables 2 – 4), we advocate for the use of explicit communication [6,34] rather than implicit expressions of uncertainty, grounded in research and logical reasoning. We encourage further exploration of this topic within veterinary medical education.

It is important to note that while this supplementary material provides examples of open-ended inquiries [21,28,36], written communication cannot fully convey the nuances of speaking pace, tone, and vocal expression, all of which play critical roles in effective communication [21,22]. This material predominantly focuses on verbal communication; however, non-verbal cues (e.g., body language, eye contact, and gestures) are also significant but have not been covered in depth due to space considerations.

Table 2. Proposed open-ended enquiries and other communication strategies useful in discussing uncertainty with the client. Note that italicized text means verbal communication.

Communication element	Example sentences	References
Assessing the effect of uncertainty	*Could you share your thoughts on what specific challenges you are facing in this case? Understanding the areas of uncertainty will help us engage in a more focused discussion and work together to clarify those elements.*	[21,25,28]
Creating a plan of management	*I understand that this situation is causing you significant discomfort and is linked to considerable economic losses. The uncertainty surrounding the definitive diagnosis is likely frustrating. It's important to recognize that the syndrome of sudden death is multifactorial, which often complicates accurate diagnosis. While we have identified the most probable diagnosis so far, we will continue to investigate this case together. Please know that I am here to support you throughout this process.*	[9,12,22,25,27,37]
	Over the next week, I will gather additional information to help us make the most informed decision regarding this situation.	
	The effect of the vaccine should be visible in about a week. If this is not a clostridial disease, then the number of deaths is not going to be controlled with the vaccination. We should be looking for signs of decreased milk production, sensitivity to sunlight, and chronic wasting in suspected cattle. It is also possible, in the next week, to see some other signs that will guide us in a different direction.	[19]
Giving reassurance	*I recognize that discussing the multifactorial nature of the sudden death syndrome, which complicates the definitive diagnosis, can be stressful. While we may not have all the answers right now, we have a clear starting point and a management plan in place. We will diligently work to uncover the underlying causes as much as possible.*	[22]
Shared decision-making	*Mr. Doe, I understand that you are facing a sudden death syndrome in your cows. It's crucial that we develop a plan to address this issue and minimize the risk of recurrence. I would like to collaborate with you to explore the best options available.*	[9,12,20,24,27,28,31]
Setting expectations	*There are instances when our knowledge and capabilities may be limited, and it's important to acknowledge that we cannot always have certainty. However, I am committed to conducting further research, seeking expert advice, and returning to you with the most informed answers possible.*	[22]
Transparent disclosure of uncertainty	*While I have strong reasons to believe this may be a case of clostridial abomasitis, I would still like to investigate further by checking for other causes of sudden death.*	[6,10,12,19,21,33,34,37,38]
	Although I cannot predict exactly when the deaths will cease, I believe that we can work together to significantly reduce the number of fatalities in the near future.	
	Currently, we do not have a clear understanding of the situation.	
	Based on the available information and our current veterinary medical knowledge, the optimal management strategy for this case remains uncertain.	
Use of safety netting/Keeping options open	*If the sudden deaths persist or increase, please do not hesitate to reach out to us. We may need to reassess the entire situation to ensure that nothing has been overlooked.*	[9,33,39]

Table 3. Proposed communication strategies for discussing uncertainty with the client using various communication competencies. Note that italicized text means verbal communication.

Communication competency	Example sentences	References
Avoid the creation of a false illusion of precision[1]	*The mortality rate among cattle affected by clostridial diseases is notably high.* rather than *Mortality in affected cattle with clostridial diseases is 65.34%.*	[25,26,32]
Delivery of uncertainty	*Currently, our working diagnosis is sudden death syndrome, which can stem from various causes. While I have strong suspicions, I remain open to exploring other possibilities as well.* rather than, *Although I am nearly certain this is a case of clostridial abomasitis, I would like us to consider other options as well.*	[9,22,33]

Eliciting the client's perspective	We can utilize the 'ICE' mnemonic to guide our discussion:<br>• **Idea:** *What do you believe is happening?*<br>• **Concerns:** *What specific worries do you have?*<br>• **Expectations:** *What outcomes were you hoping to achieve from this encounter?*	[25]
	I would like to know if this plan works for you and whether there are any potential obstacles we should consider.	[39]
Expressing empathy	*I recognize that this situation is quite challenging for you. Please know that we are here to collaborate and support you in every possible way.*	[21,28,36,40]
Expressing readiness to assist the client in a non-medical sense	*My role is not only to provide veterinary guidance but also to offer emotional support. I am here to listen if you wish to discuss anything.*	[40]
Facilitating the shared decision-making process²	*Let's work collaboratively to ensure we make the best possible decisions regarding the sudden death of your dairy cows.*	[9,12,20,24,26-28,31]
Keeping options open/Safety netting	*As previously mentioned, we suspect that this sudden death may be related to clostridial disease, but we will also remain vigilant for potential toxicity.*	[9,24,37,41]
Preparing the client for the discussion on uncertainty; Eliciting the client's idea (perspective)	*Before I share my initial thoughts on this case, I would appreciate it if you could share your perspective on what you believe is happening.*	[19,21,28,33]
Preparing the client for the discussion on uncertainty; Eliciting the client's main concerns	*We will soon explore the potential causes of the sudden deaths in your cows. However, before we delve into that, I'd like to hear about your main concerns today.*	[19,21,26,28]
Preparing the client for the discussion on uncertainty; Eliciting the client's expectations	*I want you to know that I am here to support you throughout this situation. May I ask what you were hoping to achieve from today's visit?*	[19,21,28]
Preparing the client for the discussion on uncertainty: Imperfect veterinary knowledge	*There are instances where culture results may yield negative findings while other methods confirm the presence of the causative pathogen. It's important that we all understand this before determining which tests to conduct. Are we all aligned in our understanding of the likelihood of confirming the diagnosis immediately?*	[9,22,26,33]
Preparing the client for discussion of uncertainty; the likelihood of failure of selected management option/s	*Given that sudden death syndrome can arise from multiple causes, we may need to reassess our management strategy in a few days. We will need to monitor the effects of our proposed management before revisiting our plan. Are we all in agreement on this?*	[9,26]
Preparing the client for the need for follow-up	*Due to the many unknowns in this case, it will be important to schedule further visits and discussions. During our next appointment in three days, we can focus on strategies to prevent the sudden death syndrome.*	[9,26]
Provide clear and specific instructions to the client about warning signs and syndromes that may be expected	*Since clostridial diseases often present without visible signs prior to death, we need to identify the 'high-risk cows.' Based on existing literature and your experience, we should focus particularly on high-producing cows with the highest body condition scores. Does this align with your thoughts, and could you share your reasoning?*	[19,25]
Providing emotional support to the client	*As we discussed earlier, the primary suspicion is that this sudden death may be due to clostridial disease, but we will also remain cautious about the possibility of toxicity.*	[24,25,36,37,41]
	Managing a case of sudden death is undoubtedly stressful, with complexities stemming not only from economic and emotional concerns but also from the multiple potential causes and uncertain laboratory results.	
	Regrettably, clostridial diseases in cattle result in significant losses to the industry annually.	
	It is completely natural to feel uncertain about the next steps when navigating such complex situations as the one you are currently facing.	
Providing reassurance to the client	*I will be here to support you throughout this journey, regardless of the outcome.* rather than, *While the diagnosis of the sudden deaths is not yet clear, I am confident that we can improve the situation through vaccination efforts.*	[24]
Stimulating proactive management by the client	*We should explore some proactive management strategies, as many of these approaches are likely to prevent recurrence of this issue while also enhancing overall health and production outcomes. When would you be ready to discuss this?*	[26]

¹ The client should be aware that even well-researched areas and established facts suffer from a level of randomization; ² Some clients prefer a veterinary practitioner to take the responsibility of the decision-making

Additionally, communication competency plays a crucial role at the team level (Table 4). Collaborative support is essential for effectively managing high levels of uncertainty [12,13,19,22,42,43], particularly for developing veterinary learners and professionals.

Table 4. Proposed open-ended enquiries and other communication strategies useful in discussing uncertainty with the team. Note that italicized text means verbal communication.

Communication competency	Example sentences	References
Eliciting team members' perspective	*Could you please advise me on the next steps to take in the case of sudden death in cattle that I previously mentioned?*	[22]
	I am feeling anxious about managing this situation, and any assistance you could provide would be greatly appreciated.	
Initiating a discussion regarding uncertainty	*I have an intriguing case of sudden death in cattle, and I would love to hear your insights on it.*	[12,17,22,44]

Glossary

Term	Meaning
Clinical encounter	Any physical or virtual contact with a veterinary patient and client (e.g., owner, employee of an enterprise) with a primary responsibility to carry out clinical assessment or activity
Clinical teaching	A form of interpersonal communication between a clinical instructor and a learner that involves a physical or virtual clinical encounter
Debrief	A formal and structured analysis of the action carried out in order to obtain useful intelligence or information that could be applied in future

Effective feedback	A purposeful conversation between the clinical instructor and the veterinary medical professional to stimulate further development of clinical competencies and deep learning
(Clinical) Instructor	A person who, in addition to the regular veterinary practitioner's duties, a clinical instructor should fulfil the roles of assessor, facilitator, mentor, preceptor, role model, supervisor, and teacher of veterinary learners in a clinical teaching environment. It may include any of the following: Apprentice/intern in the upper years, Resident, Veterinary educator/teacher, or Veterinary practitioner
Reflection	The metacognitive process that may occur before, during or after an encounter aims to develop a deeper understanding of the encounter and self ± the team to inform the ongoing and/or future actions, behaviors, and encounters
Reflective practice	A metacognitive, learner-centered strategy that assists learners in making sense of the learned material and engaging in deep learning
Safe (learning) environment	An environment in which a learner feels safe, relaxed, and willing to take risks in pursuing a goal; enhances self-esteem and encourages exploration
The five microskills model	An instructor-centered model of clinical teaching: 1) Get a commitment; 2) Probe for supporting evidence; 3) Teach general rules; 4) Reinforce what was done well; and 5) Correct mistakes. An additional stage is the 'Debrief'.
- Get a commitment	Encourages the learner in obtaining, processing and synthesizing of information
- Probe for supporting evidence	Based on reflective practice, it encourages the learner to give a rationale for their selected hypothesis (e.g., differential diagnosis or management strategy)
- Teach general rules	Non-essential step of the model, which is recommended to assist the learner's understanding of general medical reasoning and principles in individual encounters
- Reinforce what was done right	Based on effective feedback, provides descriptive, case-specific and behavior-focused feedback to the learner every time a task has been handled well
- Correct mistakes	Based on effective feedback, providing constructive criticism, descriptive, case-specific and behavior-focused feedback to the learner every time a task could be improved
- Debrief	Not a direct element of the Five microskills model, based on reflective practice that should assist the learner in differentiation between important and non-important aspects of the environment, and provide an opportunity for discussing the entire encounter

References

1. ACGME. Family medicine milestones. Available online: https://www.acgme.org/specialties/family-medicine/milestones/ (accessed on 14 Jan 2025).
2. Royal Australia College of General Practitioners. Managing uncertainty. *Standards for general practice, 5th Ed* **2023**, 277.
3. Gärtner, J.; Berberat, P.O.; Kadmon, M.; Harendza, S. Implicit expression of uncertainty - Suggestion of an empirically derived framework. *BMC Medical Education* **2020**, *20*, doi:10.1186/s12909-020-1990-3.
4. Carr, A.N.; Kirkwood, R.N.; Petrovski, K.R. Using the five-microskills method in veterinary medicine clinical teaching. *Veterinary Sciences* **2021**, *8*, doi:10.3390/vetsci8060089.
5. Carr, A.N.; Ferlini Agne, G.; Kirkwood, R.N.; Petrovski, K.R. Teaching clinical reasoning to veterinary medical learners with a case example. *Encyclopedia* **2024**, *4*, 753-775, doi:10.3390/encyclopedia4020048.
6. Meyer, A.N.D.; Giardina, T.D.; Khawaja, L.; Singh, H. Patient and clinician experiences of uncertainty in the diagnostic process: Current understanding and future directions. *Patient education and counseling* **2021**, *104*, 2606-2615, doi:10.1016/j.pec.2021.07.028.
7. Beck, J.B.; Long, M.; Ryan, M.S. Into the unknown: Helping learners become more comfortable with diagnostic uncertainty. *Pediatrics* **2020**, *146*, doi:10.1542/peds.2020-027300.
8. Ferlini Agne, G.; Carr, A.N.; Kirkwood, R.N.; Petrovski, K.R. Assisting the learning of clinical reasoning by veterinary medical learners with a case example. *Veterinary sciences* **2024**, *11*, 433, doi:10.3390/vetsci11090433.
9. Ingham, G. To do or not to do: Teaching the skill of deciding what to do in the face of uncertainty. *Australian journal of general practice.* **2024**, *53*, 900-903, doi:10.31128/AJGP-08-23-6949.
10. Rising, K.L.; Cameron, K.A.; Salzman, D.H.; Papanagnou, D.; Doty, A.M.B.; Piserchia, K.; Leiby, B.E.; Shimada, A.; McGaghie, W.C.; Powell, R.E.; et al. Communicating diagnostic uncertainty at emergency department discharge: A simulation-based mastery learning randomized trial. *Academic Medicine* **2023**, *98*, 384-393, doi:10.1097/ACM.0000000000004993.
11. Kerr, A.M.; Thompson, C.M. Medical students' reactions to uncertainty during clinical rotations. *Family Medicine* **2022**, *54*, 285-289, doi:10.22454/FamMed.2022.947719.
12. Gheihman, G.; Johnson, M.; Simpkin, A.L. Twelve tips for thriving in the face of clinical uncertainty. *Medical Teacher* **2020**, *42*, 493-499, doi:10.1080/0142159X.2019.1579308.
13. Moffett, J.; Armitage-Chan, E.; Hammond, J.; Kelly, S.; Pawlikowska, T. "It's okay to not know …" a qualitative

exploration of faculty approaches to working with uncertainty. *BMC Medical Education* **2022**, *22*, doi:10.1186/s12909-022-03180-6.

14. Patel, P.; Hancock, J.; Rogers, M.; Pollard, S.R. Improving uncertainty tolerance in medical students: A scoping review. *Medical Education* **2022**, *56*, 1163-1173, doi:10.1111/medu.14873.
15. Berger, S.; Krug, K.; Goetz, K. Encountering uncertainty and complexity in decision-making: An observational study of clinical reasoning among medical and interprofessional groups of health care students. *Journal of Interprofessional Care* **2023**, *37*, 262-271, doi:10.1080/13561820.2022.2061928.
16. Carr, A.N.M.; Kirkwood, R.N.; Petrovski, K.R. Effective veterinary clinical teaching in a variety of teaching settings. *Veterinary sciences* **2022**, *9*, 17, doi:10.3390/vetsci9010017.
17. Herchline, D.; Cohen, M.E.; Ambrose, M.; Hwang, J.; Kaminstein, D.; Kilberg, M.; Rosenblatt, S.; Ziemba, J.; Boyer, D. Into the unknown: Characterizing fellow uncertainty during the transition to unsupervised practice. *Journal of Graduate Medical Education* **2023**, *15*, 201-208, doi:10.4300/JGME-D-22-00221.1.
18. Moffett, J.; Hammond, J.; Murphy, P.; Pawlikowska, T. The ubiquity of uncertainty: a scoping review on how undergraduate health professions' students engage with uncertainty. *Advances in Health Sciences Education* **2021**, *26*, 913-958, doi:10.1007/s10459-021-10028-z.
19. Scott, I.A.; Doust, J.A.; Keijzers, G.B.; Wallis, K.A. Coping with uncertainty in clinical practice: a narrative review. *Medical journal of Australia* **2023**, *218*, 418-425, doi:10.5694/mja2.51925.
20. Papanagnou, D.; Ankam, N.; Ebbott, D.; Ziring, D. Towards a medical school curriculum for uncertainty in clinical practice. *Medical Education Online* **2021**, *26*, doi:10.1080/10872981.2021.1972762.
21. Petrovski, K.; McArthur, M. The art and science of consultations in bovine medicine: Use of modified Calgary – Cambridge guides. *Macedonian Veterinary Review* **2015**, *38*, 137-147, doi:10.14432/j.macvetrev.2015.05.044.
22. Meyer, A.N.D.; Giardina, T.D.; Khanna, A.; Bhise, V.; Singhal, G.R.; Street, R.L., Jr.; Singh, H. Pediatric clinician perspectives on communicating diagnostic uncertainty. *International Journal for Quality in Health Care* **2019**, *31*, G107-G112, doi:10.1093/intqhc/mzz061.
23. Han, P.K.J.; Klein, W.M.P.; Arora, N.K. Varieties of uncertainty in health care: A conceptual taxonomy. *Medical decision making* **2011**, *31*, 828-838, doi:10.1177/0272989X10393976.
24. Kerr, A.M.; Thompson, C.M.; Stewart, C.A.; Rakowsky, A. "I want them to still trust me with their child's care": A longitudinal study of pediatric residents' reactions to and communication with parents about medical uncertainty across residency. *Health Communication* **2023**, *38*, 1054-1064, doi:10.1080/10410236.2021.1991637.
25. Simpkin, A.L.; Armstrong, K.A. Communicating uncertainty: A narrative review and framework for future research. *Journal of General Internal Medicine* **2019**, *34*, 2586-2591, doi:10.1007/s11606-019-04860-8.
26. Scott, I.; Gingerich, A.; Eva, K.W. Twelve tips for clinicians dealing with uncertainty when assessing learners. *Medical Teacher* **2019**, *41*, 888-894, doi:10.1080/0142159X.2018.1494381.
27. Kim, K.; Lee, Y.M. Understanding uncertainty in medicine: Concepts and implications in medical education. *Korean Journal of Medical Education* **2018**, *30*, 181-188, doi:10.3946/kjme.2018.92.
28. Carr, A.N.; Kirkwood, R.N.; Petrovski, K.R. The art and science of consultations in bovine medicine: Use of Modified Calgary – Cambridge Guides, Part 2. *Macedonian Veterinary Review* **2023**, *46*, 5-16, doi:doi:10.2478/macvetrev-2023-0011.
29. Alam, R.; Cheraghi-Sohi, S.; Panagioti, M.; Esmail, A.; Campbell, S.; Panagopoulou, E. Managing diagnostic uncertainty in primary care: A systematic critical review. *BMC family practice* **2017**, *18*, 79-79, doi:10.1186/s12875-017-0650-0.
30. Politi, M.C.; Légaré, F. Physicians' reactions to uncertainty in the context of shared decision making. *Patient*

Education and Counseling **2010**, *80*, 155-157, doi:https://doi.org/10.1016/j.pec.2009.10.030.
31. Lee, C.; Hall, K.; Anakin, M.; Pinnock, R. Towards a new understanding of uncertainty in medical education. *Journal of Evaluation in Clinical Practice* **2021**, *27*, 1194-1204, doi:10.1111/jep.13503.
32. Han, P.K.J. Conceptual, methodological, and ethical problems in communicating uncertainty in clinical evidence. *Medical care research and review* **2013**, *70*, 14S-36S, doi:10.1177/1077558712459361.
33. Cox, C.; Hatfield, T.; Fritz, Z. How and why do doctors communicate diagnostic uncertainty: An experimental vignette study. *Health expectations : an international journal of public participation in health care and health policy* **2024**, *27*, e13957-n/a, doi:10.1111/hex.13957.
34. Cox, C.L.; Miller, B.M.; Kuhn, I.; Fritz, Z. Diagnostic uncertainty in primary care: What is known about its communication, and what are the associated ethical issues? *Family Practice* **2021**, *38*, 654-668, doi:10.1093/fampra/cmab023.
35. Xia, X.; Zeng, Y.; Pi, P.; Wu, X.; Fang, X.; Chen, J.; Zhong, Y. The effect of uncertainty training on the improvement of diagnostic ability in Chinese medical students. *Journal of Medical Education and Curricular Development* **2024**, *11*, 23821205241226818, doi:10.1177/23821205241226818.
36. Segal, E.S. Maintaining communication in a time of uncertainty. *Archives of Family Medicine* **1995**, *4*, 1066-1067, doi:10.1001/archfami.4.12.1066.
37. Santhosh, L.; Chou, C.L.; Connor, D.M. Diagnostic uncertainty: From education to communication. *Diagnosis* **2019**, *6*, 121-126, doi:10.1515/dx-2018-0088.
38. Ogden, J.; Fuks, K.; Gardner, M.; Johnson, S.; McLean, M.; Martin, P.; Shah, R. Doctors expressions of uncertainty and patient confidence. *Patient Education and Counseling* **2002**, *48*, 171-176, doi:https://doi.org/10.1016/S0738-3991(02)00020-4.
39. Hewson, M.G.; Kindy, P.J.; Van Kirk, J.; Gennis, V.A.; Day, R.P. Strategies for managing uncertainty and complexity. *Journal of General Internal Medicine* **1996**, *11*, 481-485, doi:10.1007/BF02599044.
40. Darok, M.C.; Ho, L.; Holstrom-Mercader, M.S.; Lorenz, F.J.; Freiberg, A.S.; Dellasega, C.A. How medical students make meaning of uncertainty through a novel pediatric psycho-oncology rotation. *Health Professions Education* **2024**, *10*, 17-56, doi:10.55890/2452-3011.1051.
41. Moulder, G.; Harris, E.; Santhosh, L. Teaching the science of uncertainty. *Diagnosis* **2023**, *10*, 13-18, doi:10.1515/dx-2022-0045.
42. Babenko, O.; Lee, A. Ambiguity and uncertainty tolerance and psychological needs of medical students: A cross-sectional survey. *Clinical Teacher* **2022**, *19*, doi:10.1111/tct.13523.
43. Luther, V.P.; Crandall, S.J. Commentary: Ambiguity and uncertainty: Neglected elements of medical education curricula? *Academic Medicine* **2011**, *86*.
44. Cunha, L.D.M.; Pestana-Santos, M.; Lomba, L.; Santos, M.R. Barriers and facilitators to managing uncertainty in nurses' clinical reasoning in post-anaesthesia care units: a qualitative thematic analysis. *Australian journal of advanced nursing* **2023**, *40*, 4-12, doi:10.37464/2023.404.1120.
